# Gene expression mapping of the neuroectoderm across phyla – conservation and divergence of early brain anlagen between insects and vertebrates

**DOI:** 10.7554/eLife.92242

**Published:** 2023-09-26

**Authors:** Nico Posnien, Vera S Hunnekuhl, Gregor Bucher

**Affiliations:** 1 Department of Developmental Biology, Johann-Friedrich-Blumenbach Institute, University Goettingen Göttingen Germany; 2 Department of Evolutionary Developmental Genetics, Johann-Friedrich-Blumenbach Institute, University of Göttingen Göttingen Germany; Max Planck Institute for Evolutionary Biology Germany; New York University United States

**Keywords:** brain evolution, eye evolution, mid-hindbrain boundary, archicerebrum, prosocerebrum, *Tribolium castaneum*

## Abstract

Gene expression has been employed for homologizing body regions across bilateria. The molecular comparison of vertebrate and fly brains has led to a number of disputed homology hypotheses. Data from the fly *Drosophila melanogaster* have recently been complemented by extensive data from the red flour beetle *Tribolium castaneum* with its more insect-typical development. In this review, we revisit the molecular mapping of the neuroectoderm of insects and vertebrates to reconsider homology hypotheses. We claim that the protocerebrum is non-segmental and homologous to the vertebrate fore- and midbrain. The boundary between antennal and ocular regions correspond to the vertebrate mid-hindbrain boundary while the deutocerebrum represents the anterior-most ganglion with serial homology to the trunk. The *insect head placode* is shares common embryonic origin with the vertebrate adenohypophyseal placode. Intriguingly, vertebrate eyes develop from a different region compared to the insect compound eyes calling organ homology into question. Finally, we suggest a molecular re-definition of the classic concepts of archi- and prosocerebrum.

## Background

All bilaterian animals stem from a common ancestor and their development is based on an astonishingly similar set of genes. Despite this conservation on the genetic level, bilaterian morphological diversity is overwhelming, posing major challenges to homologize body or brain regions between clades. The advent of molecular data and the revised phylogenies have re-opened old discussions and have led to new hypotheses (Martín-Durán and Hejnol, 2021). Seminal work on comparing anterior gene expression and function between mice and flies revealed an astonishing conservation such as the function of the *otd/otx* genes in anterior brain formation (Acampora et al., 1998; Leuzinger et al., 1998) and a role of *eyeless/pax6* in eye formation (Gehring, 1996). Subsequently, an impressive number of additional genetic similarities fostered the conclusion that the development of the anterior part of an animal and its brain relies on a set of conserved genes, which derived from the anterior patterning system of the last common ancestor (De Velasco et al., 2004; de Velasco et al., 2007; Gąsiorowski et al., 2021; Hartenstein and Stollewerk, 2015; Hejnol and Lowe, 2015; Hirth et al., 2003; Lichtneckert and Reichert, 2005; Lowe et al., 2003; Marlow et al., 2014; Martín-Durán and Hejnol, 2021; Martín-Durán et al., 2016; Posnien et al., 2011b; Reichert, 2005; Reichert and Simeone, 1999; Simeone et al., 1992b; Sinigaglia et al., 2013; Tomer et al., 2010). Further, new fossil evidence has been used in combination with developmental data to contribute to our view on brain evolution and homologies of body regions (e.g. Chipman and Erwin, 2017; Lan et al., 2021; Lev et al., 2022; Ortega-Hernández et al., 2017; Scholtz and Edgecombe, 2006; Strausfeld et al., 2022) and similarities of neural connectivity and function of respective brain areas have been used for suggesting homology (e.g. Bridi et al., 2020; Strausfeld and Hirth, 2013). Overall, the genetic similarity based on a common origin of the anterior brains is widely accepted (e.g. Arendt et al., 2008; Hartenstein and Stollewerk, 2015; Hirth, 2010; Nielsen, 1999) while some homology statements may represent oversimplifications and the reduced nervous systems of many clades require explanation (e.g. Hejnol and Lowe, 2015). Several issues have remained controversial because of species- or clade-specific divergence in both morphology and gene expression patterns. In this review, we revisit current homology hypotheses by integrating information on gene expression patterns from the two holometabolous insect models, that is the vinegar fly *Drosophila melanogaster* and the red flour beetle *Tribolium castaneum*. So far, fly data have been mainly used for insect–vertebrate comparisons but comprehensive comparative data have been accumulating for *T. castaneum* in the past decade. Basing conclusions on both species is important given that beetles show a more conserved mode of head development than the flies with their derived involuted larval head (Klingler and Bucher, 2022; Posnien et al., 2010b; Tautz et al., 1994). Similar expression patterns in both species will be considered conserved traits for holometabolous insects while the ancestral state for insects will have to await the accumulation of respective data in more early branching insects.

We focus on the upper part of the brain of insects (supraesophageal ganglion), which is composed of three main components that derive from different developmental units, respectively. The part derived from the intercalary segment is called the tritocerebrum, the ganglion of the antennal segment forms the deutocerebrum and the neuroectoderm anterior to the antenna gives rise to the protocerebrum (Figure 2; Ito et al., 2014; Snodgrass, 1935; Strausfeld, 1976; Weber, 1966; Westheide and Rieger, 1996). While the developmental definitions of these components are unequivocal, it is difficult to morphologically delineate them in the adult brain or to draw a clear line between the supraesophageal ganglion and the subesophageal ganglion. This is due to morphogenetic movements, the morphological fusion of the brain parts and the divergent morphological constellations of the location of the esophagus relative to the brain (Ito et al., 2014). Since most molecular comparisons are based on data from *D. melanogaster*, which has a very derived anterior development, several controversies remain with respect to the homology of insect and vertebrate brain anlagen. Specifically, the internalization of head and the brain neuroectoderm during a process called head involution (Posnien et al., 2010b; Tautz et al., 1994) casts doubts on the conservation of the underlying genetic mechanisms. In the past decades, the *T. castaneum* has become an alternative insect model system for studying a more insect-typical development (Brown et al., 2009; Klingler and Bucher, 2022; Tautz et al., 1994). Comprehensive gene expression and gene function data have been gathered regarding the formation of the head and brain, opening the possibility for a comprehensive re-assessment of homologies between vertebrates and insects (Ansari et al., 2018; Coulcher and Telford, 2012; Economou and Telford, 2009; Fu et al., 2012; Kittelmann et al., 2013; Kitzmann et al., 2017; Li et al., 1996; Oberhofer et al., 2014; Posnien and Bucher, 2010a; Posnien et al., 2011a; Posnien et al., 2011b; Schaeper et al., 2010; Schinko et al., 2008; Schroder et al., 2000; Siemanowski et al., 2015; Steinmetz et al., 2010; Yang et al., 2009a; Yang et al., 2009b).

In this review, we synthesize the findings from *T. castaneum* and *D. melanogaster* to re-evaluate ongoing discussions on the homologies of insect and vertebrate brains. We base our conclusions on the comparison of expression patterns at the end of neuroectoderm patterning, arguing that this is the phylotypic stage of the brain, where similarities are highest and not yet blurred by subsequent evolutionary diversification. Hence, our homology statements refer to the *embryonic anlagen of brain structures* from which brain structures develop rather than to homology of the final brain structures. This approach is insightful because the compared stage is similar among clades in that it consists of an essentially two dimensional epithelium that precedes the complex morphogenetic movements and tissue interactions that shape and diversify the developing brain. Vertebrate neural stem cells defined in the neuroectoderm remain in the epithelium of the neural tube while postmitotic neural cells can be migratory. Insect neural stem cells (called neuroblasts) delaminate from the neuroectodermal epithelium. They do not actively migrate but may be displaced passively by morphogenetic movements of adjacent cells and tissues. While we include selected data from other species and clades to support specific hypotheses, we do neither provide a comprehensive review on the plethora of data gained in extant organisms, nor do we discuss the valuable insights gained from the fossil record.

We provide molecular evidence that confirm some previous hypotheses on brain homologies but we also propose reconsidering a number of classic and recent suggestions. First, we argue that the protocerebrum, which emerges from the pre-antennal region, corresponds to the vertebrate fore- and midbrain and that it is of non-segmental origin (i.e. not serial homologous to trunk segments and their ganglia). Second, we argue that the parasegment boundary separating the antennal segment from the pre-antennal region corresponds to the location of the mid-hindbrain boundary (MHB) of vertebrates. To reflect this homology of position and the IHB’s function as embryonic signaling center, we suggest calling it the insect head boundary (IHB). Third, we define a region with molecular similarity to the vertebrate adenohypophyseal placodes. Fourth, we summarize key arguments for a segmental nature of the antennal segment, which has recently been questioned. Fifth, we suggest reviving the classic concepts of insect archi- and prosocerebrum as subdivisions of the protocerebrum by adopting molecular definitions by *optix/six3* and *otd/otx* expression, respectively. We speculate about the brain structures that may be derived from these brain parts and the homology of the archicerebrum with the apical organ of other animals.

Within the framework of overall conservation of gene expression in the anterior region, some striking evolutionary divergences become apparent. On the one hand, we argue that the optic lobes, which are innervated by the insect compound eyes, develop from a different region than the vertebrate eyes. This argues against simple homology of these organs but interestingly opens the possibility of homology with the insect ocelli. On the other hand, we show that several genes involved in olfactory system development are expressed in different brain regions in insects and vertebrates, indicating a non-trivial evolutionary history of olfactory organs. Hence, the molecular and cellular similarities found with respect to eyes and olfactory systems may not reflect classic homology but may rather stem from the independent recruitment of homologous cell types or gene regulatory networks from the common ancestor, that is deep homology.

## Part I: Using the patterned neuroectoderm as unit of comparison across phyla

Embryonic expression patterns have proven useful for aligning and homologizing neuroectodermal regions of protostomes with deuterostomes (Hirth et al., 2003; Posnien et al., 2011a; Reichert and Simeone, 1999; Steinmetz et al., 2010; Steinmetz et al., 2011; Tomer et al., 2010; Urbach, 2007). However, given the highly dynamic expression of developmental genes during development, choosing comparable developmental stages is paramount for meaningful comparisons. According to the hourglass model, morphology and gene expression show a high degree of divergence at the beginning of embryogenesis but later converge toward a conserved developmental stage – the phylotypic stage – before diverging again (Domazet-Lošo and Tautz, 2010; Slack et al., 1993). In insects, the elongated germband stage represents the phylotypic stage – at least with respect to the epidermal development. This stage is very conserved across arthropods with respect to morphology, molecular definition of the parasegment boundaries and the subdivision into larger embryonic regions by the Hox genes. For instance, head segments were homologized across arthropods by studying this stage (Abzhanov and Kaufman, 1999; Damen et al., 1998; Eriksson et al., 2010; Telford and Thomas, 1998).

What stage should be compared with respect to the brain of vertebrates and inseccts? In both clades, neural patterning starts in the setting of an essentially two-dimensional epithelium, the neuroectoderm. At this early stage, the first spatial patterning of the neuroectoderm takes place. Subdivision is started by morphogen signals that initiate regulatory networks, which eventually lead to the differential expression of transcription factors. Thus, the neuroectoderm is subdivided into domains, which develop into different brain structures subsequently. Specifically, in vertebrates, the first spatial patterning occurs during early gastrulation concomitant with the induction of the neural plate. At the end of gastrulation, a basic spatial subdivision of the neural plate is achieved and several neural plate organizers are established, which maintain and further refine the spatial patterns. The morphogen signals emanating from the organizers influence cellular fate and growth in the adjacent tissues. Importantly for our discussion, the MHB is one of the organizers specified during early neural plate stages (Cavodeassi et al., 2016; Rhinn et al., 2006). The expression of bHLH genes marks the specification of neural progenitors. The first bHLH genes start their expression in the neural plate (Nieber et al., 2009) which is further confirmation that the first phase of spatial patterning is finished by then.

**Figure 1.**
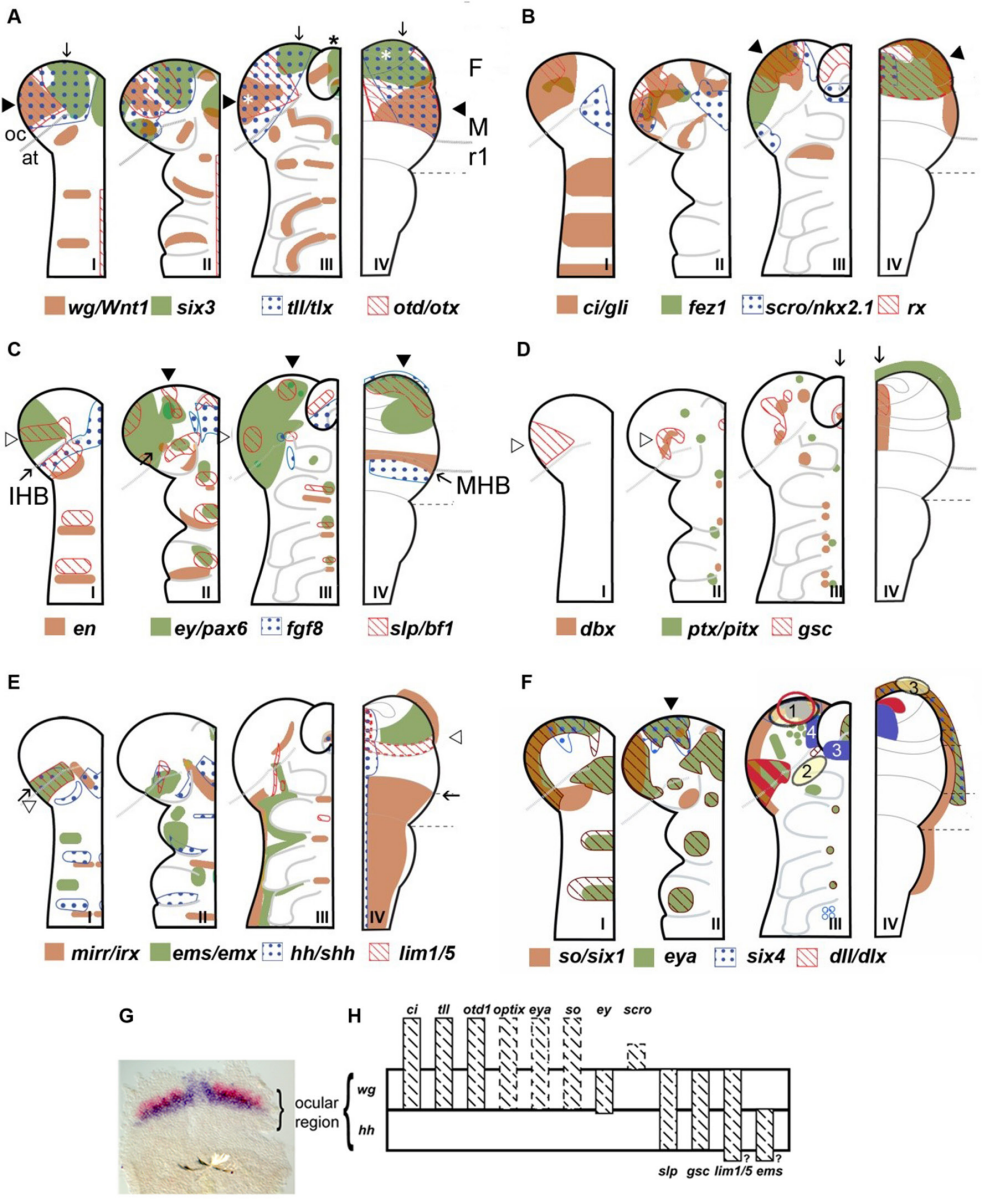
Neuroectodermal gene expression in a beetle and vertebrates. (**A–F**) Expression patterns of orthologous genes are shown for *T. castaneum* (I–III) and vertebrates (IV). The beetle data are based on Posnien et al., 2011b while the vertebrate data were compiled from several species and publications and plotted onto the schematic of a mouse late neural plate (see details below). Hence, expression boundaries are less precise for vertebrates compared to the beetle data. The stages shown for *T. castaneum* correspond to elongating germband with five trunk stripes of Tc-wingless (I), elongated germband (II), and retracting germband (III) – stage II was used for comparison with vertebrates. The changes of expression patterns reflect pattern formation processes while cell division and/or migration play a minor role if any. (**A–C**) Twelve genes show a similar arrangement of expression patterns in the anterior neuroectoderm of vertebrates and insects. Some of them are expressed almost exclusively in the anterior brain neuroectoderm in both clades while others show additionally segmental expression in insects. It seems unlikely that such a degree of similarity would arise by convergent evolution especially when considering the genes with an almost exclusive anterior expression. Together with similar findings from other animal clades we take these data as strong confirmation for the homology of the anterior neuroectoderm. However, we do not claim that homology on the level of neuroectoderm patterning will necessarily translate into clear homologies of the specific neural structures that develop from those domains. Arrowheads and arrows point to homologous regions, respectively. White stars in A depict eye anlagen. (**D, E**) Some orthologs show clearly diverged patterns. Compare posterior boundary of ems expression (white arrowhead) with location of mid-hindbrain boundary (MHB) region (arrow). (**D**) Coexpression of dbx and gsc in the stomodeum and an anterior expression of ptx/pitx (anteriormost green dot in II and III) is found in vertebrates and beetles (arrows in III and IV). Other aspects of co-expression (open arrowhead in II) and additional expression domains (e.g. ocular domain in I) have not counterpart. (**E**) Coexpression and adjacent expression of mirr/irx and *ems/emx* are found posterior to the insect head boundary (IHB) in insects but anterior to the MHB in vertebrates (compare open arrowheads relative to the arrow which marks the IHB/MHB in I and IV). Hence, the position of these domains is fundamentally different. The canonical *hedgehog* expression along the midline of the CNS in vertebrates has no correlate in insects, but medial *hedgehog* expression is found in the stomodeum. (**F**) The expression of the panplacodal genes eya, so/six1 and *six4* at the outer rim of the anterior neuroectoderm in insects and vertebrates adds further molecular similarity of bilaterian anterior patterning. Neuroendocrine tissues are marked in blue (two insect tissues are marked – PI anlagen: 4 and stomatogastric sytem: 3). The eye Anlagen in vertebrates (red) and compound eyes of insects (red hatched area) do not correspond. However, there is an anterior region which expresses many eye patterning genes and from which ocelli are likely to develop (red circle; see text and Figure 3 for more details). The Anlagen of the olfactory placode (yellow circle 3) is located at a different location than the olfactory lobes of the insect brain (yellow circle 2) but has similarity to a region, which we term insect head placode (blue circle 1). See text and Figure 3 for further details and discussion on homology of visual and olfactory systems. (**G, H**) The parasegment boundary separating the antennal segment from the ocular region (the insect head boundary, IHB) seems to be an important developmental boundary expressing the *hedgehog* and *wingless* morphogens (**G**) and marking the anterior or posterior expression boundaries of a number of patterning genes (**H**). See Supplementary file 1 for references and explanations with respect to the depiction of expression patterns. The gray dashed lines in the *T. castaneum* head indicate roughly the location of the IHB, that is the border between the ocular region (future protocerebrum) and the antennal segment (future deutocerebrum) (based on posterior border of hh expression – see text and Figure 2 for more details on the location of IHB). F: forebrain; M: midbrain; r1: rhombomere 1 of the hindbrain; open arrowhead in C I: ocular slp/bf1 domain; arrow in C II: anterior-median slp/bf1 domain; black arrowhead in C II and III: anterior-median ey/pax6 domain.

Similarly, in insects, an initially very dynamic phase of spatial patterning occurs within the neuroectodermal head epithelium (Posnien et al., 2011b; Urbach and Technau, 2003b; Urbach and Technau, 2003c). The emerging differential expression of transcription factors represents spatial information, which is then inherited by the neural stem cells, which become specified in the advanced neuroectoderm. Their specification involves the expression of bHLH genes and precedes their delamination from the neuroectoderm. Importantly, when leaving the epithelium, the insect neural stem cells ‘inherit’ the spatial information generated in the neuroectoderm (i.e. the combinatorial expression of transcription factors) and use this spatial information for assuming different identities. They are thought to obtain no further spatial information after delamination such that spatial pattern formation of the neuroectoderm is likely finalized at that stage (Skeath and Thor, 2003; Technau et al., 2006). The timing of the several waves of stem cell delamination and the expression of the stem cell marker *asense* indicate that spatial patterning has largely finished in the elongated germ-band stage (i.e. the phylotypic stage mentioned above) (panel II in Figure 1; Posnien et al., 2011b; Urbach and Technau, 2003a; Wheeler et al., 2003). This is corroborated by our expression analyses of 23 head patterning genes (Kittelmann et al., 2013; Kitzmann et al., 2017; Posnien et al., 2011b) where the initially dynamic expression converges on a more stable configuration at the elongated germband stage. Further, even the latest expression of conserved head and brain patterning genes starts shortly before that stage (Figure 1; Posnien et al., 2011b).

In summary, we believe that the late vertebrate neural plate and the insect elongated germband are valid stages for cross-phyla comparisons of brain regionalization. In the following, we will present our conclusions based mainly on comparing gene expression patterns as markers for the regionalization of the early neuroectoderm. We state homology of regions of developmental brain anlagen, which are marked by similar combinations of genes. Although the development of these regions is regulated by orthologous genes, they differ in development, morphogenesis and final morphology. Therefore, our comparison strictly remains on the level of the neuroectoderm and we refrain from homologizing the brain structures that emerge from them, in order to avoid overstatements. In each chapter, we will first present our claim and subsequently provide the underlying evidence and our reasoning.

## Part II: Conserved aspects between insect and vertebrate neuroectoderm regionalization

### Homology of the pre-antennal region to vertebrate fore- and midbrain

The comprehensive comparison of gene expression patterns in the anterior neuroectoderm of flies and beetles provides strong evidence that the insect pre-antennal region is homologous to the embryonic territory in vertebrates that gives rise to the fore- and midbrain.

Seminal work had revealed that a number of genes are active both in the vertebrate neural plate and the fly neuroectoderm. The mouse *otx* and fly *otd* proteins are so conserved that they can even rescue each other’s function (Acampora et al., 2001; Boncinelli et al., 1993; Finkelstein and Boncinelli, 1994; Leuzinger et al., 1998; Lichtneckert and Reichert, 2005; Reichert and Simeone, 1999). However, in flies, the head (i.e. the brain neuroectoderm) involutes into the body cavity, which is not typical for insects (Snodgrass, 1953) and has hampered comprehensive studies of head patterning. Therefore, we and others have studied anterior patterning in the red flour beetle *T. castaneum*, an insect species with a more insect-typical mode of head development (Posnien et al., 2010b). Expression patterns of the orthologs of most genes known to be involved in patterning the vertebrate neural plate have been determined (Posnien et al., 2011b; Schinko et al., 2008; Schroder et al., 2000; Steinmetz et al., 2011; Yang et al., 2009a). In this review, we schematically summarize the data from Posnien et al., 2011b for three stages: Before the neural stem cells (neuroblasts, NBs) start delamination (panel I), after most or all of the NBs have delaminated (panel II) and a later stage (panel III) (Figure 1). For comparison, we show the expression of the respective orthologs in a vertebrate neural plate. Note that the data for vertebrates are combined from different species at probably different stages of neural plate patterning – hence, depictions of boundaries and co-expressions are approximations and less precise compared to the beetle data (see Table S1 in Posnien et al., 2011a for references for the vertebrate genes).

Many brain patterning genes are expressed in similar patterns along the two dimensions of the vertebrate neural plate and insect neuroectoderm (Figure 1A–C) (see part III of this review for discussion of intriguing cases of non-conservation). Especially informative are conserved patterns of genes, which are expressed almost exclusively in the anterior head, because this strongly indicates that early anterior pattern was their ancestral function (*optix/six3*; *tailless/tlx*; *otd1/otx*; *earmuff/fez1*; *scro/nkx2.1*; *rx*; see Figure 1A, B). Other genes are similar in the brain anlagen but show an additional repetitive expression in the more posterior segments (*ey/pax6*; *slp/bf1*; *wg/Wnt1*; see Figure 1C). Taken together, these data confidently map the anlagen of fore- and midbrain of vertebrates to the pre-antennal region of insects which is in line with previous work in flies (Hirth et al., 2003; Urbach, 2007).

Work with different subsets of these genes in other taxa such as centipede, annelid, hemichordate, invertebrate chordate, and sea urchin confirmed the conservation of an anterior set of patterning genes but will not be discussed here in more detail (Holland and Short, 2008; Holland and Takahashi, 2005; Lowe, 2008; Lowe et al., 2003; Martín-Durán and Hejnol, 2021; Martín-Durán et al., 2016; Range and Wei, 2016; Steinmetz et al., 2011; Tomer et al., 2010). *foxQ2* was not included in the scheme despite being one of the highly conserved anterior genes because, intriguingly, it was lost in the lineage leading to amphibians and mammalians (Chevalier et al., 2006; Kitzmann et al., 2017; Marlow et al., 2014; Sinigaglia et al., 2013; Yu et al., 2003).

### The *insect head boundary* – related to the vertebrate *mid-hindbrain boundary* at the anterior boundary of the antennal segment

#### The IHB – a signaling center related to the MHB

**Figure 2.**
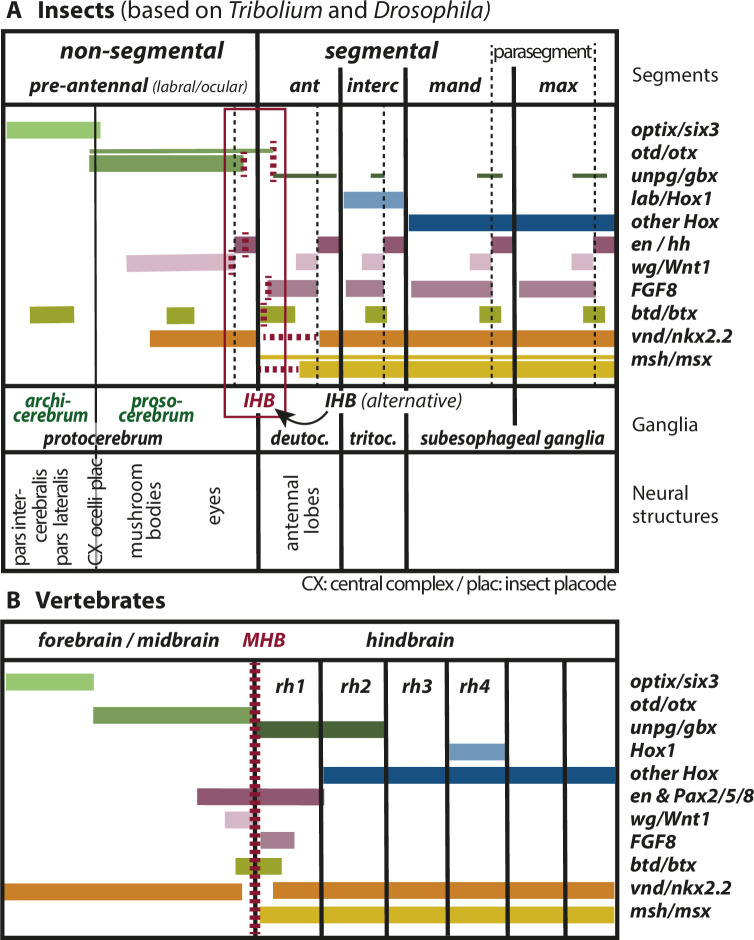
Mapping the insect head boundary (IHB) based on expression patterns of mid-hindbrain boundary (MHB) marker gene orthologs. Comparison of gene expression patterns between insects (**A**) and vertebrates (**B**) along the anterior–posterior axis. The insect data are based on patterns found in both *T. castaneum* and *D. melanogaster* in the early neuroectoderm around the time of delamination of neuroblasts (i.e. the endpoint of neuroectoderm patterning), which is comparable to the late neural plate stage shown in B. (A) Expression in the insect neuroectoderm is based on *T. castaneum* and *D. melanogaster* data. Where the data diverge or are not available for *T. castaneum*, the *D. melanogaster* pattern is indicated by thin lines (adopted from Urbach, 2007). For each gene, we indicated at what position (vertical red broken lines) or in what region the IHB would be located (horizontal red broken lines) when using only that gene as marker. Considering all the evidence (i.e. the location of all red broken lines) the data place the IHB to an area covering the interface of ocular region and antennal segment (see red box). We find no support for the alternative hypothesis that puts the IHB to the deutocerebral/tritocerebral boundary (Hirth, 2010), that is one segment more posterior (see text for discussion). Ganglia are depicted as segmental structures (see Supplementary file 1 for arguments). The segment boundaries (bold black lines) are offset relative to the embryonic parasegmental units (broken black lines shown for the subesophageal ganglia). For simplicity of the schematic, the parasegment boundaries are not marked in the more anterior segments but they are defined by adjacent non-overlapping wg/hh expression, respectively. We propose that the classic concept of protocerebral subdivision into an anterior archicerebrum and posteriorly adjacent prosocerebrum can be re-defined by *optix/six3* and *otd/otx* expression, respectively (green font). The more posterior trunk ganglia are similar to the subesophageal ganglia. Based on *six3* and *otd* expression, we assign the neuroendocrine pars intercerebralis and pars lateralis (PI/PL) to the archicerebrum and the eyes to the prosocerebrum. The mushroom bodies (MB) are likely part of the prosocerebrum while we tentatively assign the central complex (Cx), the ocelli and the insect head placode (plac) to a mixed origin. The data are combined from single and double stainings of different sources (Beermann and Schröder, 2008; Posnien et al., 2011b; Schinko et al., 2008; Steinmetz et al., 2011; Urbach, 2007; Wheeler et al., 2005) such that not all data (e.g. of FGF8, vnd, and msh) are based on a precise mapping at the same stage. Hence, the locations of some expression boundaries are approximations. See Supplementary file 1 for more details on the reasons for the mapping. Expression of genes involved in MHB formation and function in vertebrates (redrawn from Urbach, 2007). Given the difference of the gene regulatory networks subdividing insect segments and vertebrate rhombomeres, respectively, we hesitate to claim homology of antennal segment with rhombomere 1 despite the fact that both are devoid of *otd* and Hox gene expression (see text for discussion on the tripartite brain). More generally, we do not homologize segments and rhombomers.

**Figure 3.**
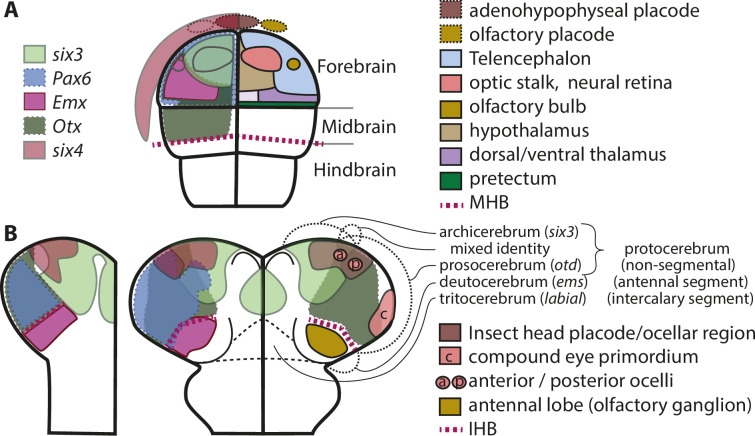
Molecular fate maps of insect and vertebrate neuroectoderm questions simple homology of eye and olfactory organs The expression domains of selected patterning genes are depicted in the left halves of a schematic mouse neural plate (**A**) and in beetles (**B**). The beetle data represent two different stages of development corresponding to I and II of Figure 1B. A simplified fate map of the anterior neural plate is shown in the right half in A (for optic and olfactory parts, the positions are outlined in the left half as well). The right half shown in B depicts the expression of the markers of archi- and prosocerebrum *six3* and *otd* (identical to left half) in addition to the approximate position of the anlagen of visual and olfactory neuropils. This molecular mapping shows that brain parts used for olfaction do not derive from the same region of the neuroectoderm in vertebrates and insects (compare yellowish shapes in A with B). Likewise, the larval insect eyes (which will develop into the adult compound eyes) do not derive from a region corresponding to the vertebrate eye anlagen (compare red shapes in A with red shape marked with c in B). Hence, for both olfaction and compound eye, the homology hypothesis is strongly questioned based on the criterion of position. Intriguingly, the ocelli emerge at positions much more similar to the vertebrate situation. This opens the possibility of homology of vertebrate eyes with ocelli (especially the *six3* positive anterior ones) but a claim of homology would need further testing. The similarity of gene regulatory networks that have been found between vertebrates and insects may be based on either co-option of networks or on conserved cell types that were assembled independently to form organs with similar function. The patterns are approximations and the exact location of expression domain boundaries or tissue anlagen remain to be determined by dedicated experiments (see details below and in the text).

**Figure 4.**
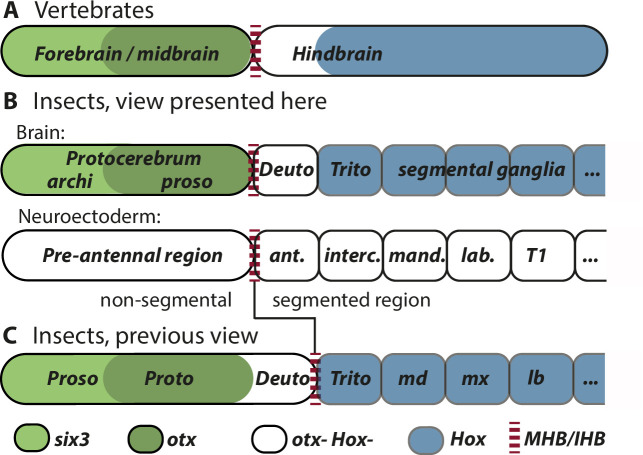
Alternative views on the subdivisions of the brain anlagen. Mapping of brain regions by using expression patterns at the neuroectodermal stage from vertebrates (neural plate) (**A**) and insects (elongated germband stage) (**B**, **C**). For insects, alternative interpretations are shown (B versus C). In both clades, the anterior expression of *six3/optix* is followed by a domain with *otx/otd* expression while the posterior is marked by the expression of Hox-cluster genes. A region expressing neither *otx/otd* nor Hox genes is separating these domains. (A) In vertebrates, the mid-hindbrain boundary (MHB) emerges at the interface between *otx* positive and Hox gene negative cells. Note that the six3 expression limit does not reflect the boundary between fore- and midbrain anlagen. (B) View presented in this paper partially based on Urbach, 2007. Both, the subdivisions of the brain (top row) and the subdivisions of the neuroectoderm (lower row) are shown. In line with classic literature comparing insect and annelid brains, the protocerebrum is suggested to be subdivided into an anterior archicerebrum and a posteriorly adjacent prosocerebrum. We suggest *six3/optix* and *otd* to be markers for these regions, respectively. The antennal segment is considered serially homologous to trunk segments. Being the anterior-most segment and being the only segment free of Hox gene expression, it might have distinctive properties. The parasegment boundary separating the non-segmental pre-antennal (or ocular) region from the antennal segment represents the insect head boundary (IHB). Its location matches the one of the MHB of vertebrates (red broken lines). We hesitate to make strong statements of homology between vertebrates and insects for the region posterior to the IHB/MHB anlagen. The data supporting this view are combined from flies and beetles. (C) Alternative view based on Hirth et al., 2003, which was recently updated by Bridi et al., 2020 and Strausfeld et al., 2022. In this view, the antennal region is not a segment – the intercalary segment is the anterior-most serially homologous trunk segment. The region corresponding to the MHB is located at the boundary between antennal region and the intercalary segment. See text for arguments supporting the view shown in B and Supplementary file 1 for discussion on possible reasons for diverging interpretation.

We argue that the boundary between the ocular region and the antennal segment of insects corresponds in position to the anlagen of the vertebrate MHB (Figure 1, Figure 2, Figure 3). This resolves conflicting interpretations of fly data, where some have suggested the same position (Urbach, 2007) while others have suggested a location one segment more posterior (Bridi et al., 2020; Hirth et al., 2003; Strausfeld et al., 2022; Figure 4).

When comparing expression patterns relative to segments, it is important to realize the offset that has been observed between the developmental boundaries of the early embryo and the later morphologically visible segment boundaries. In case of the morphological segments, the posterior boundary of *engrailed* expression represents the molecular marker. However, the embryologically relevant boundaries are defined by the interface of *wingless* and *hedgehog* expression, which coincides with the anterior boundary of *engrailed* expression. Hence, the early embryonic boundaries are offset relative to the later morphological boundaries and have been called parasegment boundaries (compare black dotted lines of parasegment boundaries with black lines depicting segment boundaries in Figure 2; Martinez-Arias and Lawrence, 1985).

The region separating antennal from pre-antennal regions shows an early expression similar to parasegment boundaries, that is the adjacent expression of the morphogens *wingless* and hh. Actually, this is the first parasegment boundary to become visible and remains the most anterior one (Figure 1G). This boundary seems to have an important embryonic signaling function in insects because of the early expression of these two morphogens. A bit later FGF-8 becomes expressed posterior to that boundary adding another morphogen signaling to that region (Figure 2; Beermann and Schröder, 2008; Farzana and Brown, 2008; Nagy and Carroll, 1994; Oberhofer et al., 2014; Posnien et al., 2011b). In line with the hypothesis of an important signaling function in early head and brain patterning, 9 out of 18 genes investigated in Posnien et al., 2011b start to be expressed in stripes parallel to the IHB (*otd*, *tll*, *lim1/5*, *gsc*, *ci*, *slp*, *ems*, *ey*) (Posnien et al., 2011b) and later, several genes have either their anterior or posterior expression boundary in that region (Figure 1H). Indeed, the activation of some of those genes was shown to depend on *wingless* and/or *hedgehog* signaling (*tailless*; *eyeless/pax6*; *twin of eyelss*; *sloppy paired1/brain factor*; *goosecoid*) while this was not found for others (*Tc-lim1*, *otd1*, and *ems*) (Oberhofer et al., 2014). Because that study focused on a very early stage, the germ rudiment (Figure 1G) later effects on target gene expression may have been missed. Taken together, the region homologous to the MHB has important embryonic signaling function. Apart from the fact that MHB and IHB are signaling centers at a homologous position we do not claim that the specific functions are the same. Hence, the known functions of the MHB such as the patterning dopaminergic neurons in the midbrain will likely not be found at the IHB. To indicate homology of location of the anlagen but likely divergence in signaling function, we suggest calling the area the *insect head boundary* (Figure 2).

Our mapping of the IHB based on the homology of fore- and midbrain anlagen to pre-antennal regions (see above) aligns with the view from Urbach, who analyzed fly data at comparable neuroectodermal stages (Figure 4B; Urbach, 2007). An alternative hypothesis based on fly data puts the MHB homolog one segment more posteriorly to the antennal/intercalary parasegment boundary (Figure 4C; Bridi et al., 2020; Hirth, 2010; Hirth et al., 2003). The location of the IHB strongly influences hypotheses on homologies between vertebrate and insect brains and some recent conclusions were based on the latter model (Bridi et al., 2020; Strausfeld et al., 2022). Therefore, we will discuss the data from flies and beetles in more detail to justify our diverging view on the matter.

#### Flies: Is the IHB located at the anterior or the posterior of the antennal segment?

In their seminal study in flies, Hirth et al. found that in insects like in vertebrates, the anterior part of the central nervous system is marked by expression of *otd/otx* orthologs, the posterior part by Hox gene expression and in between there is an intervening zone (IZ) devoid of either marker. Based on this, they suggested that the tripartite organization described in vertebrate brain was ancestral in all bilaterians. This pattern of gene expression has been confirmed for many animal taxa and likely represents the ancestral urbilaterian state (Castro et al., 2006; Hirth, 2010; Hirth et al., 2003; Holland and Short, 2008; Pani et al., 2012; Smith et al., 2018; Steinmetz et al., 2011; Steinmetz et al., 2011; Urbach, 2007; Wollesen et al., 2015). It should be noted that several early branching ecdysozoans lack a tripartite morphology such that the tripartite organization may also have evolved independently (Hejnol and Lowe, 2015). In vertebrates, the MHB emerges in the anterior IZ. It is specified at the developmental boundary defined by adjacent non-overlapping expression of *otx* and *gbx* (Rhinn et al., 2005) and influences patterning of the adjacent regions, that is the posterior midbrain and the anterior hindbrain. *Pax2/5/8* genes mark the MHB and the first rhombomere of the hindbrain represents large part of the IZ because it is free of Hox gene expression (Kiecker and Lumsden, 2005; see Figure 2B). Using *otd*, *gbx*, and *Pax2/5/8* as markers at the embryonic stage 13/14, Hirth et al. mapped the IHB to the deutocerebral/tritocerebral boundary (i.e. the antennal/intercalary segment boundary; see Figure 2; Hirth et al., 2003).

Urbach re-analyzed the expression of these genes and included a number of additional MHB markers. He used the late neuroectoderm for his investigation (see reasoning above) (Urbach, 2007) to map the region homologous to the MHB (i.e. what we call IHB) to the boundary between antennal and ocular segments, that is one segment more anterior than the previous suggestion but in line with our suggestion (Figure 2, see above). Besides adjacent *otd* and *unpg/gbx* expression, *wingless* and *buttonhead* were used as markers that have their anterior-most segmental stripe-like expression around the ocular/antennal boundary. It should be noted that the respective *engrailed* domain deviates from the expectation in that it does not form a stripe but is reduced to a ‘head spot’ (Brown et al., 1994; Patel et al., 1989). However, stripe like expression at the IHB of other arthropods indicates that this is an insect-specific deviation (Damen, 2002; Patel et al., 1989). Further, the expression of columnar genes (i.e. genes that are expressed along the body axis and are involved in animal neural patterning) reflects the findings from vertebrates: Vertebrate *Nkx2.2* is expressed along the body axis but in the MHB region this stripe is interrupted. Similarly, its fly ortholog *ventral nerve cord defective* (*vnd*) shows ventral expression domains in all segments except for the antennal segment. Vertebrate *msx* has its anterior expression boundary at the MHB and its fly ortholog *msh* is expressed up to the pre-antennal/antennal boundary. Hence, the location of the IHB at the ocular/antennal boundary is backed by data from several genes belonging to two independent patterning systems – the dorso-ventral and the anterior–posterior patterning systems. In vertebrates, *FGF8* expression marks the region posterior to the MHB. In flies however, the respective orthologs *pyramus* and *thisbe* seem not to be expressed in that region (Urbach, 2007). The main differences to the previous work is that Urbach et al. studied an earlier developmental stage (i.e. the phylotypic stage), he used more genes to map the MHB homolog and his re-analysis of *shaven/Pax2* expression led to a different conclusion with respect to its expression in the brain. In Supplementary file 1, we discuss in more detail these reasons for the divergent interpretations.

#### Beetle data confirm the location of the IHB at the anterior boundary of the antennal segment

Data from *T. castaneum* confirm and complement the results from Urbach (Figure 2; Birkan et al., 2011; Nie et al., 2001; Posnien and Bucher, 2010a; Posnien et al., 2011b; Schinko et al., 2008). The antennal segment represents the IZ, which is devoid of both, *otd* and Hox gene expression (Figure 2). Assuming a similar position at the anterior of the IZ one would place the IHB to the anterior antennal segment. In contrast to flies, the single FGF-8 ortholog in *T. castaneum* is expressed as expected from vertebrate data in the developing antennal segment and has its anterior border a bit posterior to the ocular/antennal boundary (Beermann and Schröder, 2008; Posnien et al., 2011b). Beetle *engrailed* expression is a more unequivocal marker then it is in flies because in the latter there are additional idiosyncratic expression domains anterior to the pre-antennal/antennal boundary, which had been interpreted as additional segment boundaries (Schmidt-Ott and Technau, 1992) until data from more insects showed that this is not a conserved pattern. The segmental expression of *wingless* and *buttonhead* is similar to *D. melanogaster* including non-segment-like aspects of expression in the pre-antennal region (Brown et al., 1994; Nagy and Carroll, 1994; Schinko et al., 2008). The columnar genes seem to accord as well (Wheeler et al., 2005) but a more exact mapping of their expression in the head region would be desirable to strengthen that claim. To our knowledge, the expression of *PoxN* has not been published for *T. castaneum* such that we cannot test the findings from *D. melanogaster* (see above).

Taken together, data from fly and beetle neuroectoderm place the IHB at the interface of ocular region and antennal segment (see red box in Figure 2A) although the exact mapping would vary depending on the genes used for the comparison (red dashed lines in Figure 2). In our view, the protocerebrum corresponds to the vertebrate fore- and midbrain and the deutocerebrum relates to anterior Hox-free part of the vertebrate hindbrain, which is represented by rhombomere 1 in vertebrates (Kiecker and Lumsden, 2005). However, we do not want to homologize the antennal segment with rhombomere 1 apart from the fact that both emerge from a Hox-free neuroectodermal region. We hypothesize that the IHB separates protocerebrum from deutocerebrum. Of note, *otd* expression seems not to extend into the antennal segment in *T. castaneum*, the hemipteran *Oncopeltus fasciatus* (Birkan et al., 2011), while this seems to be the case in flies (Figure 2).

### The *insect head placode* and its evolutionary connection to the vertebrate cranial placodes

We propose that a region in the anterior insect head termed the *insect head placode* shares an evolutionary origin with the vertebrate cranial placodes. This claim is based on a careful study of coexpression of a number of orthologs of vertebrate panplacodal markers and the finding that these genes mark a morphologically distinguishable structure at a similar anterior-median position. These data are summarized here but extensively presented in Posnien et al., 2011a.

The vertebrate cranial placodes contribute to a variety of cell types of the neuroendocrine system, and of sensory neurons of the olfactory, visual, and acoustic systems. They develop from non-neural ectoderm surrounding the anterior neural plate (Figure 3A; Baker and Bronner-Fraser, 2001; Brugmann and Moody, 2005; Grocott et al., 2012; Litsiou et al., 2005; Schlosser, 2010; Schlosser, 2014). The adenohypophyseal and olfactory placodes are the anterior-most placodes (Figure 3A), followed posteriorly by the lens, trigeminal, otic, and other placodes (Brugmann and Moody, 2005; Streit, 2007). Nearly, all placodal cells and their anlagen are marked by the expression of *six1* (*sine oculis*), *six4*, and *eyes absent*, which have been termed panplacodal markers (Ghanbari et al., 2001; Pandur and Moody, 2000; Schlosser, 2014; Schlosser and Ahrens, 2004). It has long been assumed that cranial placodes are vertebrate inventions (Gans and Northcutt, 1983) but an earlier evolutionary origin has been discussed as well and scenarios for the evolution of the vertebrate-specific placodes from pre-existing developmental regions and gene regulatory networks have been provided (Glenn Northcutt, 2005; Posnien et al., 2011a; Schlosser, 2005; Schlosser, 2015; Schlosser, 2017).

In flies, the formation of neuroendocrine and other neural structures via several morphologically visible placodes has been described. Further, a molecular similarity of the fly *pars intercerebralis* with the vertebrate hypothalamus has been noted based on the expression of several marker genes (*nkx2.1/vnd*; *sim1/sim*; *rx/Drx*; *six3/optix*) (de Velasco et al., 2007). In a different approach in beetles, we have identified an embryonic region that seems evolutionary related to the *vertebrate cranial placodes* (sensu *strictu*). Based on position relative to the brain anlagen, co-expression of all panplacodal markers and markers for neuroendocrine cells we defined a region as the *insect head placode*. Note that the placodes described in de Velasco et al., 2007 were defined by their morphology while the *insect head placode* is defined by its molecular similarity to vertebrate cranial placodes. The marked region was found at an anterior-median position relative to the neuroectoderm, which is similar to the vertebrate situation. Specifically, *six1/so* and *eya* are expressed in a stripe around the rim of the *T. castaneum* brain neuroectoderm (Figure 1F). Later, this co-expression splits into two domains, one at the anterior-most rim and one in the ocular region (Figure 1D, II). *six4* marks only the anterior co-expression domain (Figure 1D). The preplacodal marker *Dll/Dlx3/5* is co-expressed as well (Figure 1D). Of note, the *insect head placode* becomes morphologically distinguishable from the adjacent epidermal tissue. Further, we found a number of markers for neuroendocrine tissues being expressed there (Posnien et al., 2011a): *optix/six3* (Steinmetz et al., 2011; Tessmar-Raible et al., 2007), *pitx1* (Tremblay et al., 1998), *bf1/sloppy paired* (Choe and Brown, 2007; Toresson et al., 1998), and *eyeless/pax6* (Li et al., 1994; Schlosser and Ahrens, 2004; Yang et al., 2009a). Importantly, the specific adenohypophyseal placode markers *pitx* and *lim3/lhx3* are expressed in the *insect head placode* (Dutta et al., 2005; Pommereit et al., 2001; Posnien et al., 2011b; Taira et al., 1993). Further, *vsx/chx* and *fas2*, which are markers for the developing insect neuroendocrine *pars intercerebralis*, are expressed there (de Velasco et al., 2007; Posnien et al., 2011b).

Based on the extensive similarity of co-expressions, the *insect head placode* is likely to have evolved from the same developmental region from which the vertebrate placodes evolved. Alternatively, the similarity could stem from an ancestral co-expression in neuroendocrine cell types, which were recruited to form a structure at an anterior position (Posnien et al., 2011a; Schlosser, 2015). The most specific marker for the *insect head placode* is *six4* and the molecular and positional similarity is highest with the adenohypophyseal placodes of vertebrates, from which the anterior pituitary gland develops. The pituitary gland is innervated by the hypothalamus, the neuroendocrine brain center of vertebrates. Structurally similar, the insect *pars intercerebralis* connects to the insect neuroendocrine gland *corpora cardiaca*. While both respective structural units in vertebrates and insects contain neurosecretory cells, their exact evolutionary relationships are under discussion (Hartenstein, 2006; Tessmar-Raible, 2007). In addition, a similar molecular profile of hypothalamic cells and anterior neurosecretory cells has also been found between zebrafish and the annelid *Platynereis*, corroborating a common evolutionary origin of anterior neuroseretory cell groups. Developmental data from the beetle show that a contribution of the *six4* positive *insect head placode* to insect neuroendocrine tissue of the *pars intercerebralis* is very likely. In addition, *six4* in *Drosophila* is also a marker for type 2 neuroblast derived lineages (Chen et al., 2021) which produce the columnar cells of the *pars intercerebralis* that innervate the central complex (Boyan and Reichert, 2011). The location of type 2 neuroblasts and the six4 positive tissue may well coincide but the spatial and temporal emergence of type 2 neuroblast lineages has yet to be determined in the beetle. In contrast, a close relationship of the *insect head placode* to the olfactory placode is questionable as insect olfaction occurs mainly in the antennal segment and the expression of *ems/emx*, a crucial marker of olfactory tissues, is found in the antennal segment but not in the anterior neural tissue as in vertebrates (Figure 1; see discussion below; Posnien et al., 2011b; Schinko et al., 2008). It would be interesting to further determine, which cell types develop from the *insect head placode* and to what extent they perform similar functions compared to the cells of the pituitary and/or to what degree different cell types and functions emerge from this homologous patterning region. It remains enigmatic, in how far the insect head placode functionally relates to vertebrate placodes and if homologous cell types can be identified in this region.

### Deutocerebrum and the antennal segment – anterior-most serial homologs

We argue in favor of the classic view that the antennal segment and the deutocerebrum represent the anterior-most segment and the ganglion derived from it, respectively (e.g. Damen et al., 1998; Lev et al., 2022; Posnien et al., 2010b; Rempel, 1975; Scholtz and Edgecombe, 2006; Telford and Thomas, 1998). While this view had been common sense for a long time, it has recently been challenged by the suggestion that the antennal segment and the deutocerebrum derive from the non-segmental anterior part of the embryo. As corollary of that view, the intercalary segment forming the tritocerebrum is considered the anterior-most segment with serial homology to trunk segments (Strausfeld et al., 2022). In a comment to that paper, based on molecular and morphological data from onychophorans and fossil data it was suggested that all parts of the brain are segmental in nature including the homologs of the insect antennal segment and ocular/pre-antennal parts (Budd et al., 2023). Yet another recently proposed view is that neither antennal and intercalary segments should not be considered serially homologous because they develop by a different genetic mechanism (Lev et al., 2022). We use the term *segment* to define a structure, which is serially homologous to trunk segments. Given the early stages under scrunity for our comparison we use here the expressions of genes that define the segment boundaries as markers (i.e. *wingless*, *hedgehog*, and *engrailed*).

In support of serial homology to the trunk segments, the antennal segment is bounded by two molecularly defined parasegment boundaries, which are defined by adjacent stripes of *wingless* and *hedgehog* expression, respectively (Figure 2; Martinez-Arias and Lawrence, 1985; Ntini and Wimmer, 2011b; Oberhofer et al., 2014; Schmidt-Ott and Technau, 1992). The most famous marker of posterior segment boundaries is *engrailed*. In most insects (apart from *D. melanogaster*, which shows additional anterior domains), the anterior-most neuroectodermal *engrailed* domain emerges at the parasegment boundary separating antennal and pre-antennal region (i.e. at the IHB). In insects, this anterior-most domain is not a stripe but a ‘head spot’ (Brown et al., 1994; Patel et al., 1989). Given that in Crustaceans, spiders and centipedes the respective domain forms a segment-like stripe (at least at advanced embryonic stages), the head spot seems to be an insect-specific deviation from an ancestral segmental pattern (Damen, 2002; Kettle et al., 2003; Scholtz et al., 1994; Sintoni et al., 2007). Similar to *engrailed*, several other genes with segmental expression have their anterior-most segment-like domain in the antennal segment. These genes include *upgd/gbx*, *empty spiracles*, and *gooseberry-d* (Dalton et al., 1989; Schinko et al., 2008; Urbach, 2007; Urbach and Technau, 2003b) and the columnar gene *msh* (Urbach, 2007; Wheeler et al., 2005). Further support comes from an extensive analysis of neuroblast marker genes. For almost all tritocerebral neuroblasts, a homolog with similar combinations of gene expressions could be identified in the more posterior segments, and this is true for about half of the neuroblasts of the antennal segment. Conversely, the authors found not much if any homology of protocerebral neuroblasts to those of the trunk segments (Urbach et al., 2016). Hence, the insect neural stem cells reflect a segmentally reiterated pattern including the antennal segment but not the more anterior pre-antennal region. Of course, there are segment-specific deviations from the ground pattern not only in the antennal segment such as the reduction of neuroblasts in the intercalary and other segments (Urbach et al., 2016). A completely different type of evidence shows the segmental nature of the antennal segment in the most striking way: When the entire Hox-cluster is deleted in beetles, all trunk segments carry antennae (Stuart et al., 1991). Such a result indicates that the basic genetic subdivision into segments (i.e. the definition of the segment boundaries, their anterior–posterior and their dorso-ventral sub-patterning) is very similar between antennal and more posterior segment.

In our view, the ocular region does not represent a serially homologous segment. The main argument is that the ocular region lacks an anterior parasegment boundary that would separate it from the non-segmental anterior head tissue. However, its posterior boundary is indeed defined in segment-like way by the IHB, which shows adjacent expression of *wingless* and *hedgehog* like canonical parasegment boundaries as well.

Apart from these similarities supporting serial homology of the antennal with the more posterior trunk segments, there are idiosyncrasies, which may derive from being the anterior-most segment. Most importantly, the antennal segment shows neither *otd* nor Hox gene expression (see discussion on the tripartite brain above) thus representing the intervening zone of the tripartite brain (see above). Intriguingly, we found three marker genes, which are expressed early and rather exclusively in the antennal segment of beetles distinguishing it from both, trunk segments and pre-antennal region: *ems; lim1/5* and *goosecoid* (Posnien et al., 2011b). A similar *empty-spiracles* pattern was found in a centipede as well (Hunnekuhl and Akam, 2017). Moreover, *empty-spiracles* and FGF8 show an antenna-specific expression before similar segmental patterns emerge (Posnien et al., 2011b). While the similarity of half of the antennal neuroblasts argues for a segmental nature (see above), the other half appears to have diverging identities, possibly regulated by the above-mentioned antennal-specific genes, indicating quite some divergence as well.

Intriguingly, the formation of the antennal parasegment boundary is different from the one of trunk segments: It involves a dynamic splitting of the ocular *hedgehog* stripe in two stripes, the posterior of which becomes the antennal hh-stripe. This was found in both *O. fasciatus* and *T. castaneum* (Lev and Chipman, 2021 and own data). The posterior adjacent intercalary segment of insects is formed yet by a different mechanism (Crozatier et al., 1999; Ntini and Wimmer, 2011a; Posnien and Bucher, 2010a). Actually, *hedgehog*-stripe splitting was first found in spiders (Kanayama et al., 2011) and Centipedes (Hunnekuhl and Akam, 2017), but in these clades one additional stripe splitting is observed, which results in the formation of the posterior boundary of the segment, which is homologous to the intercalary segment, respectively. The different gene regulatory dynamics leading to the formation of these segments has been taken as evidence for non-homology of those segment with trunk segments. Further, it was claimed that they evolved after the trunk segments in an independent way, secondarily adopting segment-like characters (Lev and Chipman, 2021; Lev et al., 2022). It is out of the scope of this work to discuss the evolution of segmentation. However, we propose that the structural, cellular, and molecular similarities of mature segments are strong arguments for serial homology despite the different developmental processes. Indeed, in some insects, the anterior segments form simultaneously while the posterior ones are added sequentially (Stahi and Chipman, 2016). Nevertheless, the resulting segments are regarded serially homologous.

Taken together, we suggest the antennal segment being the anterior-most of the serially homologous trunk segments. Some degree of divergence is found in many segments but the antennal-specific aspects and their relevance for deutocerebrum development may stem from its position at the segmental/non-segmental interface and are worth dedicated analyses.

### Archicerebrum and prosocerebrum revisited

#### New molecular code for classic concepts

We propose to revive and re-define the classic concepts of archicerebrum and prosocerebrum to denominate subdivisions of the insect protocerebrum. These subdivisions correspond to the prostomial and peristomial brain parts of annelid larvae and are defined by expression of *optix/six3* and *otd/otx*, respectively. These markers were chosen because they subdivide the anterior brains across animal phyla indicating an ancestral function (Steinmetz et al., 2010).

Inspired by comparisons with annelids, the insect protocerebrum was suggested to consist of two parts. By some authors, the anterior archicerebrum was defined as the ganglion of the acron, which is the anterior-most non-segmental part of insect head. The acron in turn was thought to be homologous to the larval episphere and the adult prostomium of annelids, respectively. In that view, the archicerebrum was suggested to derive from the apical organ of the last common ancestor of annelids and insects (e.g. Snodgrass, 1935; Westheide and Rieger, 1996). The posteriorly adjacent prosocerebrum was often considered to be the ganglion of the first serially homologous trunk segment, identified as the ocular segment by many authors. However, several alternative views have been put forward as well and no consensus has emerged on which parts of the insect brain actually represent the archi- or the prosocerebrum. For example, an alternative topology was recently discussed where the prosocerebrum represents the most anterior brain part and the archicerebrum is either assigned to a subterminal lateral brain region including the eyes (Steinmetz et al., 2010) or is omitted as a concept altogether (Strausfeld et al., 2022). These diverging views reflect the uncertainties with respect to the number of head segments, the disputed segmental nature of the labral region and the extent of non-segmental tissue in the insect head (Chaudonneret, 1950; Posnien et al., 2010b; Remane, 1950; Rempel, 1975; Rogers and Kaufman, 1997; Schmidt-Ott and Technau, 1992; Scholtz and Edgecombe, 2006; Siewing, 1963; Weber, 1966; Westheide and Rieger, 1996).

A molecular subdivision into an anterior labral part and a posterior ocular part of the brain was formulated based on segment polarity and columnar gene expression (Urbach and Technau, 2003b). A slightly different subdivision was subsequently proposed based on the expression of *optix/six3* and *otd/otx* (Steinmetz et al., 2010). We suggest following the latter subdivision and using that molecular code for re-defining the assignment of brain structures denominated by the terms archi- and prosocerebrum (Figure 2 and Figure 4). The core argument for adopting the latter molecular subdivision is that the anterior neuroectoderm of many if not all bilaterian animals is partitioned into an anterior *optix/six3* positive tissue and a more posterior *otd/otx* marked region (insects, centipedes, annelids, vertebrates, sea urchins, hemichordates, and others) (Carl et al., 2002; Lowe et al., 2003; Steinmetz et al., 2010; Wei et al., 2009). Further, in a single-cell sequencing approach in spiders, *six3* and *otd* expressing cells fell into clearly separated clusters with the former marking a more anterior tissue than the latter (Leite et al., 2022). In most studied animals, these two genes belong to the earliest expressed markers of anterior neuroectoderm and a repressive effect of Wnt signaling on *six3* expression seems to be widely conserved as well. This is also true for *Tribolium*, where only few other markers are expressed at a similarly early stage in the protocerebrum anlagen. Among these, *tailless* covers both *six3* and *otd* regions, *foxQ2* is expressed in the *six3* domain while *eyeless/pax6* marks the posterior-most part of the *otd* marked protocerebrum (Kitzmann et al., 2017; Posnien et al., 2011b). Taken together, these molecular markers suggest an ancient early role of *optix/six3* and *otd/otx* in subdividing the anterior neuroectoderm of bilaterian animals (Steinmetz et al., 2010). It should be noted that the expression of *optix/six3* and *otd/otx* is dynamic during development and not mutually exclusive at all stages. Indeed, brain parts of mixed origin are expected to emerge from the region of overlap (see Figure 2 and below).

The molecular definition of archi- and prosocerebrum leads to some corollaries, which deviate from previous definitions. First, according to our data, both archi- and prosocerebrum are non-segmental (i.e. not derived from serial homologs of trunk segments) while classically, the prosocerebrum was thought to derive from the anterior-most segment (Remane, 1950; Strausfeld, 2012; Weber, 1966). Our claim of the non-segmental nature is based on the molecular similarity of the entire insect pre-antennal region to the fore- and midbrain of vertebrates and our mapping of the IHB (Figures 1 and 2; see above). Importantly, the annelid orthologs of the key markers *optix/six3* and *otd/otx* are expressed in the pro- and peristomium, respectively, which are undisputed non-segmental areas in these protostomes (Posnien et al., 2011b; Steinmetz et al., 2011; Tomer et al., 2010; Urbach, 2007). As second corollary, no labral or ocular segment exist in the insect head making the antennal segment the anterior-most segment and the deutocerebrum the anterior-most ganglion, which are serially homologous to trunk segments (see above). Finally, the *six3*-based definition of the archicerebrum comprises a larger domain than the previously suggested labral subdivision of the protocerebrum (Urbach and Technau, 2003b), which was defined based on segment polarity and columnar gene expression. Actually, the latter subdivision is a sub-part of the *six3* domain.

#### Brain parts belonging to archi- or prosocerebrum or both

The molecular definition of archi- and prosocerebrum allows for hypothesizing of brain structures emerging from the respective neuroectodermal regions (see Figure 3). The archicerebral *six3* marked embryonic domain comprises the developing neuroendocrine *pars intercerebralis* and *pars lateralis* as shown by expression of the markers chx and fasciclin2 within the *six3* positive region in *D. melanogaster* and *T. castaneum* (de Velasco et al., 2007; Posnien et al., 2011b; Steinmetz et al., 2010). In *T. castaneum*, the expression of these markers is deleted in RNAi knocking down *six3* (Posnien et al., 2011b).

The mushroom bodies are probably part of the prosocerebrum based on two lines of evidence. The mushroom body neuroblasts were molecularly mapped in fly (Kunz et al., 2012) and in a separate study the same authors mapped *six3* expression in brain neuroblasts (Steinmetz et al., 2010). Comparing these two maps, the MB neuroblasts seem to lie outside of the *six3* domain. RNAi data confirm this to some degree: RNAi knock-down of *six3* in beetles has not much effect on mushroom body development (Posnien et al., 2011b). However, RNAi targeting *foxQ2* (which is expressed in the *six3*region) leads to aberrant mushroom bodies but not to their loss (He et al., 2019; Kitzmann et al., 2017). Assuming a prosocerebral origin of the MBs, these defects would have to be interpreted as indirect effects, which is well possible but remains to be shown.

The assignment of the insect ocelli is difficult. Their position on the anterior-median head and the location of at least some cell bodies of secondary neurons contributing to the ocellar tract and nerves in close to or within the *pars intercerebralis* indicate a connection with the archicerebrum (Figure 3; Mizunami, 1995a; Mizunami, 1995b). However, in flies, the anlagen of all ocelli in the eye-imaginal disc are *otd* positive and require the *otd* gene for their development indicating relation to the prosocerebrum (Domínguez-Cejudo and Casares, 2015; Finkelstein et al., 1990). Interestingly, only the anlagen of the anterior ocelli show co-expression of *otd* with *six3* and require *six3* function (Domínguez-Cejudo and Casares, 2015). Taken together, data from the rather derived fly situation would indicate a prosocerebral origin for the lateral and a mixed origin for the anterior ocelli. In the grasshopper *Schistocerca gregaria* with its more insect-typical development, the ocelli emerge from an anterior patterning field positive for *eyes-absent* and *sine-oculis* (Dong and Friedrich, 2005). Morphologically and based on co-expression of *eyes-absent* with *six4*, this embryonic region is similar if not identical to the insect head placode (IHP) as defined in *T. castaneum* (see above). In line with the mixed origin seen in flies, only the anterior part of the IHP is marked by *six3*, while the posterior is marked by *otd*. In the future, one will have to test the hypothesis that the insect head placode corresponds to the ocellar region in flies, and that this region is of mixed archicerebral and prosocerebral origin. Unfortunately, *T. castaneum* does not have ocelli and the fly ocelli develop from the highly diverged imaginal disks such that this question needs to be addressed in other species. The hemimetabolan cricket *Gryllus bimaculatus*, the grasshopper *S. gregaria*, or the bug *O. fasciatus* are excellent candidates to test this hypothesis.

We propose that the CX is built from both, archi- and prosocerebrum for the following reasons: At least four type II neuroblasts contributing to the WXYZ tracts of the central complex are located in the pars intercerebralis of grasshoppers, hence, they likely derive from the archicerebrum (Boyan and Reichert, 2011; Boyan and Williams, 1997). Further, we found that knock-down of *six3* leads to the deletion of the central complex in beetles (Posnien et al., 2011b). Similarly, *foxQ2* is initially co-expressed with *six3* (i.e. archicerebral) and in beetles, *foxQ2* positive neurons contribute to the CX and its knock-down led to aberrant CX development (He et al., 2019; Kitzmann et al., 2017). On the other hand, *rx* seems to be largely or even exclusively co-expressed with *otd* (Figure 1) but there is strong contribution of *rx*-positive neurons to both, columnar and tangential neurons of the CX of beetles and flies (Davis et al., 2003; Farnworth et al., 2020) and lack of *rx*-function led to aberrant CX development in beetles (Koniszewski, 2011) and flies (Davis et al., 2003). Hence, strong prosocerebral contribution to the CX is justified just as well. Actually, a mixed origin is likely given that the CX consists of columnar and tangential neurons, which have a basically orthogonal direction of projections into that neuropil (Boyan and Reichert, 2011; Loesel et al., 2002; Pfeiffer and Homberg, 2014; Strausfeld, 1976). For instance, the columnar neurons derive from DM1–4 neuroblasts, which map to the anterior-median region (Walsh and Doe, 2017), that is probably the archicerebrum as defined here. Conversely, at least some *ellipsoid body* tangential neurons stem from a neuroblast that emerges in the *engrailed* head spot (i.e. the prosocerebrum as defined here) and forms the EB-A1/DALv2 neural lineage (Bridi et al., 2019; Omoto et al., 2018).

Given the difficulty comparing data between taxa and across publications, dedicated studies in relevant model systems are needed to test these hypotheses. Besides work based on the power of the *D. melanogaster* model system, new genome editing techniques may eventually allow genetically marking the neurons derived from defined areas of the neuroectoderm in emerging model insects such as *T. castaneum* and the hemimetabolan *G. bimaculatus*.

#### The archicerebrum – emerging from a region homologous to the apical organ?

Expression of the molecular markers *six3* and *foxQ2* in the archicerebral embryonic region suggests that this part of the insect embryo develops from a neuroectodermal region homologous to the apical plate of primary marine larvae. Centered within the apical plate, most ciliated protostome and deuterostome larvae (such as phoronids, brachiopods, echinoderms, and enteropneusts) and cnidarian larvae have an apical organ. This organ is a sensory and neuroendocrine structure comprised of early born neurons, neurosecretory cell types, sensory neurons, light sensitive cells, and ciliated cells, which may form an apical tuft (Marlow et al., 2014; Nielsen, 2005). Accordingly, taxa without primary larvae (such as arthropods or vertebrates) show no homologous structure to the apical tuft (Nielsen, 2005). Based on morphological data, no clear homologous structure has been found in insects nor vertebrates and it was suggested that, in marine invertebrates, the neural part of the apical organs may degenerate during metamorphosis (Nielsen, 2005). However, a similar anterior gene regulatory network (aGRN) specifies the anterior-most parts of embryos across animal phylogeny. This aGRN includes *six3*, *foxQ2*, *rx*, *nkx2.1*, and other genes and its activation requires repression of Wnt signaling coming from the posterior. In many animals, *six3* seems to be the upstream component of the aGRN, which by mutual repression with Wnt signaling first defines the area, which is subsequently subdivided by mutual interactions among the aGRN genes (Carl et al., 2002; Darras et al., 2011; Fritzenwanker et al., 2014; Leclère et al., 2016; Lowe et al., 2003; Marlow et al., 2014; Momose et al., 2008; Oliver et al., 1995; Sinigaglia et al., 2013; Steinmetz et al., 2010; Tessmar-Raible et al., 2007; Wei et al., 2009; Yaguchi et al., 2008; Yaguchi et al., 2016). Based on this set of conserved regulators expressed in homologous regions (i.e. the anterior tip of the animal), molecular homology of this neuroectodermal region is very likely. While the cell types of the apical organ of some ciliated larvae have been described (see Marlow et al., 2014 and references cited therein), a dedicated study of potentially homologous cell types in insects has been missing. Candidates for homologous cell types in insects are the *pars intercerebralis* and/or *pars lateralis* or the *insect head placode*. Another intriguing possibility for homologous cells are early born pioneering neurons contributing to the anteriormost arthropod brain. In the grasshopper *S. gregaria*, such neurons emerge within the aGRN epidermal region and are required for the first steps of midline crossing (Boyan and Williams, 2008). In a centipede, which is a mandibulate arthropod well suited as an outgroup to insects, early born pioneer neurons with epithelial origin that might be homologous to the short-range pioneers in the grasshopper, develop from the *six3*/*foxQ2* positive region. In addition, these neurons express apical organ markers (Hunnekuhl and Akam, 2014). This parallels observations in annelids, where some early born apical epidermal neurons are important for the early brain development (e.g. Lacalli and Berrill, 1981; Meyer et al., 2015; Tessmar-Raible et al., 2007). It should be noted that the regulation by orthologous genes does not imply homology of the structure. Therefore, in depth morphological and developmental-genetic comparative studies seem warranted to reveal the cellular and functional similarity versus divergence emerging from a clearly homologous patterning field.

## Part III: Divergent patterns with relevance for the homology of eyes and olfaction

### Genetic interactions are not conserved to a large degree

The similarity of the gene sets patterning the anterior neuroectoderm is striking and its conservation over hundreds of millions of years requires explanation. One reason could be that the regulatory interactions of the conserved genes constrain their expression, such that even small changes would have dramatic effects. We found that in *Tribolium*, *six3* represses *Wnt1* similar to findings in mouse, sea urchin, and annelids (Lagutin et al., 2003; Lavado et al., 2008; Marlow et al., 2014; Wei et al., 2009). Moreover, *Xpax6* is activated by *Xsix3* in *Xenopus* (Gestri et al., 2005), which is also true for the probably homologous mushroom body domain of *ey* but not for the probably non-homologous early ocular domain (Posnien et al., 2011b). However, we also found profound differences: *Xotx* and *Xrx-1* expression are reduced in *Xsix3* knock-down embryos in *Xenopus* (Gestri et al., 2005) in contrast to *Tribolium*, where *otd* expands and *rx* does not change (Posnien et al., 2011b). Most strikingly, *FoxQ2* is on the one hand a very conserved gene of the aGRN but on the other hand has been lost completely in amphibians and mammalians. Further, while it is an aGRN downstream component in some animals, it has gained an upstream function in beetles (Chevalier et al., 2006; Hunnekuhl and Akam, 2014; Kitzmann et al., 2017; Ogawa et al., 2021; Sinigaglia et al., 2013; Yaguchi et al., 2008; Yu et al., 2003). In summary, despite the similarity of expression, the genetic interactions seem to be conserved only to minor degree. Hence, the observed conservation of the gene set may not derive so much from early regulatory interactions during pattern formation but rather to conserved later functions, for example in specifying cell types (Arendt, 2008; Tessmar-Raible et al., 2007) or by co-option of gene regulatory networks leading to ‘homocracy’ of structures (i.e. regulation by the homologous genes) without indicating classic homology (i.e. derived from a common ancestor organ; Nielsen and Martinez, 2003).

The limited level of regulatory conservation contrasts the impressive degree of biochemical conservation of the involved genes. For instance, it has been shown that mouse and fly otx/*otd* and *ems/emx* orthologs can rescue the respective mutants of the other clade, respectively (Lichtneckert et al., 2008 and references therein). This shows a striking conservation of biochemical function, which has been used as argument for claiming conserved function in the formation of the tripartite brain. Conversely, it has been argued before that conservation of the biochemical function of proteins does not mean conservation of the structures that are formed from them (Hejnol and Lowe, 2015). Given that we see fundamentally different location of for example *ems/emx* expression between vertebrates and insects (Figure 1), it seems that biochemical functional conservation of a transcription factor is no strong argument for homology of structures.

### Vertebrate eyes: homolog to insect ocelli?

We argue that the location of the compound eye anlagen is fundamentally different from the anlage of the vertebrate eyes (Figure 3). This questions the homology on the organ level despite molecular and cellular similarities. Our arguments therefore support the previous notion that eyes evolved several times in parallel from ancestral light sensory cell types and their circuits. Hence, the molecular and cellular similarities reflect deep homology as coined by Shubin et al., 1997; Shubin et al., 2009 rather than homology on the organ level. We note that the insect ocelli are anterior visual organs that – at least based on their anterior-median position – represent alternative candidates for being homologs of the vertebrate eyes.

The evolution of the eyes has remained a controversial issue. Since the finding of *eyeless/pax6* as a key upstream component of eye development in insects and vertebrates, the homology versus the convergent evolution of visual organs from ancestral cell types has been discussed intensively (Arendt, 2003; Arendt et al., 2004; Arendt et al., 2009; Erclik et al., 2009; Friedrich, 2022; Gehring, 1996; Gehring, 2005; Gehring and Ikeo, 1999; Hartenstein and Reh, 2002; Sen et al., 2013; Vopalensky and Kozmik, 2009; Wagner, 2007). On the one hand, homology of vertebrate eyes and insect compound eyes was suggested based on the involvement of *eyeless/pax6*, the six family of homeobox genes and other genes in the respective gene regulatory networks and of additional homologous genes being required for the development of respective interneurons (e.g. Arendt, 2003; Gehring, 2005; Hartenstein and Reh, 2002). Further, homologous location of respective primordia in *otd/otx* and tailless/tlx co-expressing tissues anterior to the Hox-marked trunk was used to homologize eyes across taxa. Together with similarities of the neurogenetic mechanisms used for eye formation, homology of the developmental mechanisms of fly optic lobe/compound eye with vertebrate tectum/eye formation was suggested (Erclik et al., 2008; Erclik et al., 2009; Joly et al., 2016). Alternatively, molecular and morphological similarity of certain cell types was interpreted as homology on the level of cell types and it was argued that the visual organs and their retinal axonal circuits may have been assembled independently from ancestral cell types (Arendt et al., 2004; Arendt et al., 2009; Erclik et al., 2009; Nilsson and Arendt, 2008), a view supported by others who pointed out the differences in the eye specification networks (Wagner, 2007). Such an evolutionary scenario is suited to explain *deep homology*, that is the finding of molecular similarities in diverged or even non-homologous morphological structures (Shubin et al., 1997; Shubin et al., 2009).

Our data show that the vertebrate eye and the insect compound eye emerge from different regions of the neuroectoderm strongly questioning homology on the level of the organ of these eyes. The region of the vertebrate neural plate giving rise to the eyes and olfactory bulbs has been well defined by fate-mapping experiments (Fernández-Garre et al., 2002). The embryonic origin of insect eyes and olfactory brain parts (i.e. antennal luobes) has been mapped molecularly (Das et al., 2008; Dong and Friedrich, 2005; Snodgrass, 1935). The vertebrate eye anlagen are located medio-laterally in the anterior neural plate (red in Figure 3A; Inoue et al., 2000; Puelles et al., 2005; Rubenstein et al., 1998; Shimamura et al., 1995). In contrast, in grasshopper and beetles, the compound eye develops from a region between two ocular *wingless* domains, which arise by splitting of the early ocular stripe (brown shape in Figure 1A, I and II shows split of ocular *wingless*-domain; red shape in Figure 3B depicts eye anlagen) (Dong and Friedrich, 2005; Liu et al., 2006; Yang et al., 2009a; Yang et al., 2009b). Within the similarity of the molecular framework discussed above, these are clearly non-homologous positions (compare Figure 3A with B). It should be noted that the position of the visual anlagen in *D. melanogaster* seem to emerge from a different location (Chang et al., 2001; Friedrich, 2013). In Supplementary file 1, we discuss, why we think that the *D. melanogaster* situation may be derived. In summary, our data point to an independent evolution of insect compound eyes. The genetic similarity would then stem from ancestral visual cell types independently recruited to both types of organs in line with previous suggestions of deep homology (Shubin et al., 2009).

Intriguingly, the insect ocelli emerge in an anterior-median region of the neuroectoderm not unlike the vertebrate eye anlagen (red shapes in Figure 3A, B). While unfortunately, *T. castaneum* does not have ocelli, we find several eye markers (*optix/six3*, *scro/nkx2.1*, *rx*, *ey/toy/Pax6*, and *slp/bf1*) expressed in both, the anlagen of the compound eyes and in an anterior-medial part of the neuroectoderm, that is the IHB (Figures 1 and 3). This opens the possibility that the last common ancestor had light sensory organs developing from the anterior-median region of the neuroectoderm, evolving into ocelli in insects and into the vertebrate eyes, respectively. Based on fossil evidence, it has been argued that the median eyes/ocellar systems represent the primordial visual system that evolved before the emergence of compound eyes (Schoenemann and Clarkson, 2023). Hence, in the lineage leading to the insects, the lateral complex eyes might have developed as evolutionary novelty by activating the ancestral eye specification program at a non-homologous position. Indeed, the misexpression of only one transcription factor is sufficient for the formation of functional ectopic eyes that project into the nearest ganglion in flies (Clements et al., 2008; Halder et al., 1995). This event might have happened early in arthropod evolution as spider eyes emerge from two different regions as well: The principle eyes, which are located anterior-median (similar to ocelli) and the secondary lateral eyes, which in horseshoe crabs show similarity to insect compound eyes (Friedrich, 2022; Samadi et al., 2015; Schomburg et al., 2015).

This scenario may contribute to explaining differences of the eye specification network, which have led to the suggestion of an independent evolution of the eyes (Shubin et al., 2009; Wagner, 2007). Specifically, in vertebrates, *rx* is crucial to initiate eye development and the network requires *six3*/6 function. In contrast, *rx* is not involved in fly compound eye development and a curious switch was observed where *six3*/6 was replaced by another family member, the *six1/2* ortholog *sine-oculis*. According to our mapping, *six3* and *rx* are expressed in close vicinity albeit in probably non-overlapping patterns in the insect head placode, which probably represents the region of ocelli development (compare Posnien et al., 2011a with Posnien et al., 2011b). The anterior fly ocelli express *six3* (Domínguez-Cejudo and Casares, 2015) and we suggest that *rx* expression in the lateral ocelli seems possible based on our data (although this remains to be shown).

Taken together, the fundamentally different location of the eye anlagen within the early neuroectoderm means that the *homology criterion of similar position* is not fulfilled. This strongly suggests that the insect compound eyes are not homologous to the vertebrate eye – at least on the organ level. However, the fact that several orthologous genes are involved in the development of vertebrate and insect compound eyes suggests that homology might be found on other levels. For instance, the cell types and/or the genes that determine them could well be derived from ancestral light sensory cell types (Arendt et al., 2016; Erwin, 2020; Shubin et al., 2009). In that view, the compound eye evolved independently from vertebrate eyes by assembly of ancestral gene regulatory networks and/or cell types at different positions. Intriguingly, the location of the ocelli anlagen at the anterior and the nearby expression of several genes required for vertebrate eye development actually imply that the ocelli are better candidates to look for homology on the level of visual organs. Unfortunately, *D. melanogaster* shows a derived head development (i.e. in imaginal discs) while *T. castaneum* has lost the ocelli. Hence, a hemimetabolan model system with an embryonic origin of ocelli might be most adequate to test this possibility.

### Orthologs of *Emx*, *Lhx5*, and *goosecoid* mark olfactory organs at non-homologous sites in insects versus vertebrates

Similar to the eyes, we find that the anlagen of the olfactory brain regions are located at very different positions relative to the conserved anterior patterning region (Figure 3). Hence, simple homology of the olfactory organs on the organ level seems unlikely.

In vertebrates, the anlagen of the optic stalk are located lateral and anterior to the eye anlagen in the anterior neural plate (Figure 3A). In contrast, the insects olfactory organ emerges from the antennal segment (Figure 3B). Interestingly, the expression of several genes involved in olfactory development reflect these different positions. *empty-spiracles* is a very specific early marker for the antennal segment in *T. castaneum* and *O. fasciatus* (Birkan et al., 2011; Posnien et al., 2011b; Schinko et al., 2008) while in *D. melanogaster*, it marks several adjacent segments (Cohen and Jürgens, 1990) and is required for the postembryonic development of olfactory circuits (Lichtneckert et al., 2008; Sen et al., 2013). In vertebrates, *emx* paralogs are expressed in the anlagen of the olfactory system and in double mutants, the olfactory bulb is severely affected (Mallamaci et al., 1998; Shinozaki et al., 2002; Simeone et al., 1992a). Likewise, *lhx5* is expressed in vertebrate olfactory anlagen (Moreno et al., 2003) and its beetle orthologs is expressed in the early antennal segment (Posnien et al., 2011b). Finally, *goosecoid* expression extends from ocular expression into the anterior antennal segment while in vertebrates it is expressed in the anterior neuroectoderm and is required for olfactory organ development among other functions (Yamada et al., 1997).

Similar to the argument discussed for the eyes, the fundamentally different location of the anlagen of the olfactory organs within the early neuroectoderm contradicts the homology of these structures on the organ level. Careful mapping of respective cells and functional data are required to test this possibility.

## Outlook

The expression of orthologous developmental genes has provided valuable arguments for homology assessments. However, evolutionary divergence blurs the signal such that comparisons need to be based on data from several taxa. Now, comprehensive data are available for two holometabolous insects, leading to some robust conclusions such as the non-segmental part of the brain, homologs of the vertebrate cranial placodes and the location of the MHB homolog. Comparable data from other arthropods and protostomes would be highly welcome to test, if our conclusions were biased by holometabola-specific aspects of expression.

A number of hypotheses presented here merit more attention and further work. For instance: What cell types develop from the insect head placode and in how far do they correspond to the adenohypophyseal cell types? In how far does the independent integration of homologous cell types explain similar gene regulatory networks in different locations of the insect compound and vertebrate eye as well as the olfactory systems? And do we find molecular and morphological similarity between insect ocelli and vertebrate eyes to claim homology and how do these eyes relate to those of crustaceans and spiders?

As argued in this work, we find the molecular similarities in the anterior neuroectoderm convincing enough to claim homology between vertebrates and insects at least at the level of early patterning of the neuroectoderm. However, we remain hesitant with homologizing specific brain structures such as the insect mushroom bodies or central complex with vertebrate structures. For the time being, we also refrain from homologizing the insect ventral nerve cord and the vertebrate spinal cord.

## Additional files

Supplementary file 1. Discussion of additional aspects and explanations for the data shown in the schematics.

## References

[bib1] Abzhanov A, Kaufman TC (1999). Homeotic genes and the arthropod head: expression patterns of the labial, proboscipedia, and Deformed genes in crustaceans and insects. PNAS.

[bib2] Acampora D, Avantaggiato V, Tuorto F, Barone P, Reichert H, Finkelstein R, Simeone A (1998). Murine Otx1 and *Drosophila* otd genes share conserved genetic functions required in invertebrate and vertebrate brain development. Development.

[bib3] Acampora D, Gulisano M, Broccoli V, Simeone A (2001). Otx genes in brain morphogenesis. Progress in Neurobiology.

[bib4] Ansari S, Troelenberg N, Dao VA, Richter T, Bucher G, Klingler M (2018). Double abdomen in a short-germ insect: Zygotic control of axis formation revealed in the beetle *Tribolium castaneum*. PNAS.

[bib5] Arendt D (2003). Evolution of eyes and photoreceptor cell types. The International Journal of Developmental Biology.

[bib6] Arendt D., Tessmar-Raible K, Snyman H, Dorresteijn AW, Wittbrodt J (2004). Ciliary photoreceptors with a vertebrate-type opsin in an invertebrate brain. Science.

[bib7] Arendt D (2008). The evolution of cell types in animals: emerging principles from molecular studies. Nature Reviews. Genetics.

[bib8] Arendt D, Denes AS, Jékely G, Tessmar-Raible K (2008). The evolution of nervous system centralization. Philosophical Transactions of the Royal Society of London. Series B, Biological Sciences.

[bib9] Arendt D., Hausen H, Purschke G (2009). The “division of labour” model of eye evolution. Philosophical Transactions of the Royal Society of London. Series B, Biological Sciences.

[bib10] Arendt D, Musser JM, Baker CVH, Bergman A, Cepko C, Erwin DH, Pavlicev M, Schlosser G, Widder S, Laubichler MD, Wagner GP (2016). The origin and evolution of cell types. Nature Reviews. Genetics.

[bib11] Baker CV, Bronner-Fraser M (2001). Vertebrate cranial placodes I. Embryonic induction. Developmental Biology.

[bib12] Beermann A, Schröder R (2008). Sites of Fgf signalling and perception during embryogenesis of the beetle Tribolium castaneum. Development Genes and Evolution.

[bib13] Birkan M, Schaeper ND, Chipman AD (2011). Early patterning and blastodermal fate map of the head in the milkweed bug *Oncopeltus fasciatus*. Evolution & Development.

[bib14] Boncinelli E, Gulisano M, Broccoli V (1993). Emx and Otx homeobox genes in the developing mouse brain. Journal of Neurobiology.

[bib15] Boyan GS, Williams JLD (1997). Embryonic development of the pars intercerebralis/central complex of the grasshopper. Development Genes and Evolution.

[bib16] Boyan GS, Williams JLD (2008). Evidence that the primary brain commissure is pioneered by neurons with a peripheral-like ontogeny in the grasshopper Schistocerca gregaria. Arthropod Structure & Development.

[bib17] Boyan GS, Reichert H (2011). Mechanisms for complexity in the brain: generating the insect central complex. Trends in Neurosciences.

[bib18] Bridi JC, Ludlow ZN, Hirth F (2019). Lineage-specific determination of ring neuron circuitry in the central complex of *Drosophila*. Biology Open.

[bib19] Bridi JC, Ludlow ZN, Kottler B, Hartmann B, Vanden Broeck L, Dearlove J, Göker M, Strausfeld NJ, Callaerts P, Hirth F (2020). Ancestral regulatory mechanisms specify conserved midbrain circuitry in arthropods and vertebrates. PNAS.

[bib20] Brown SJ, Patel NH, Denell RE (1994). Embryonic expression of the single Tribolium engrailed homolog. Developmental Genetics.

[bib21] Brown SJ, Shippy TD, Miller S, Bolognesi R, Beeman RW, Lorenzen MD, Bucher G, Wimmer EA, Klingler M (2009). The red flour beetle, Tribolium castaneum (Coleoptera): a model for studies of development and pest biology. Cold Spring Harbor Protocols.

[bib22] Brugmann SA, Moody SA (2005). Induction and specification of the vertebrate ectodermal placodes: precursors of the cranial sensory organs. Biology of the Cell.

[bib23] Budd GE, Mayer G, Janssen R, Eriksson BJ (2023). Comment on “The lower Cambrian lobopodian *Cardiodictyon* resolves the origin of euarthropod brains.”. Science.

[bib24] Carl M, Loosli F, Wittbrodt J (2002). Six3 inactivation reveals its essential role for the formation and patterning of the vertebrate eye. Development.

[bib25] Castro LFC, Rasmussen SLK, Holland PWH, Holland ND, Holland LZ (2006). A Gbx homeobox gene in amphioxus: insights into ancestry of the ANTP class and evolution of the midbrain/hindbrain boundary. Developmental Biology.

[bib26] Cavodeassi F, Moreno-Mármol T, Hernandez-Bejarano M, Bovolenta P, Castelli-Gair Hombría J), Bovolenta P (2016). Organogenetic Gene Networks: Genetic Control of Organ Formation.

[bib27] Chang T, Mazotta J, Dumstrei K, Dumitrescu A, Hartenstein V (2001). Dpp and Hh signaling in the *Drosophila* embryonic eye field. Development.

[bib28] Chaudonneret J (1950). La morphologie céphalique de *Thermobia domestica* (Packard) (Insecte Aptérygote Thysanoure). Annales Des Sciences Naturelles. Zoologie et Biologie Animale.

[bib29] Chen R, Hou Y, Connell M, Zhu S (2021). Homeodomain protein Six4 prevents the generation of supernumerary *Drosophila* type II neuroblasts and premature differentiation of intermediate neural progenitors. PLOS Genetics.

[bib30] Chevalier S, Martin A, Leclère L, Amiel A, Houliston E (2006). Polarised expression of FoxB and FoxQ2 genes during development of the hydrozoan Clytia hemisphaerica. Development Genes and Evolution.

[bib31] Chipman AD, Erwin DH (2017). The Evolution of Arthropod Body Plans: Integrating Phylogeny, Fossils, and Development-An Introduction to the Symposium. Integrative and Comparative Biology.

[bib32] Choe CP, Brown SJ (2007). Evolutionary flexibility of pair-rule patterning revealed by functional analysis of secondary pair-rule genes, paired and sloppy-paired in the short-germ insect, Tribolium castaneum. Developmental Biology.

[bib33] Clements J, Lu Z, Gehring WJ, Meinertzhagen IA, Callaerts P (2008). Central projections of photoreceptor axons originating from ectopic eyes in *Drosophila*. PNAS.

[bib34] Cohen SM, Jürgens G (1990). Mediation of *Drosophila* head development by gap-like segmentation genes. Nature.

[bib35] Coulcher JF, Telford MJ (2012). Cap’n’collar differentiates the mandible from the maxilla in the beetle Tribolium castaneum. EvoDevo.

[bib36] Crozatier M, Valle D, Dubois L, Ibnsouda S, Vincent A (1999). Head versus trunk patterning in the *Drosophila* embryo; collier requirement for formation of the intercalary segment. Development.

[bib37] Dalton D, Chadwick R, McGinnis W (1989). Expression and embryonic function of empty spiracles: a *Drosophila* homeo box gene with two patterning functions on the anterior-posterior axis of the embryo. Genes & Development.

[bib38] Damen WGM, Hausdorf M, Seyfarth EA, Tautz D (1998). A conserved mode of head segmentation in arthropods revealed by the expression pattern of Hox genes in A spider. PNAS.

[bib39] Damen WGM (2002). Parasegmental organization of the spider embryo implies that the parasegment is an evolutionary conserved entity in arthropod embryogenesis. Development.

[bib40] Darras S, Gerhart J, Terasaki M, Kirschner M, Lowe CJ (2011). β-catenin specifies the endomesoderm and defines the posterior organizer of the hemichordate Saccoglossus kowalevskii. Development.

[bib41] Das A, Sen S, Lichtneckert R, Okada R, Ito K, Rodrigues V, Reichert H (2008). *Drosophila* olfactory local interneurons and projection neurons derive from a common neuroblast lineage specified by the empty spiracles gene. Neural Development.

[bib42] Davis RJ, Tavsanli BC, Dittrich C, Walldorf U, Mardon G (2003). *Drosophila* retinal homeobox (drx) is not required for establishment of the visual system, but is required for brain and clypeus development. Developmental Biology.

[bib43] De Velasco B, Shen J, Go S, Hartenstein V (2004). Embryonic development of the *Drosophila* corpus cardiacum, a neuroendocrine gland with similarity to the vertebrate pituitary, is controlled by sine oculis and glass. Developmental Biology.

[bib44] de Velasco B, Erclik T, Shy D, Sclafani J, Lipshitz H, McInnes R, Hartenstein V (2007). Specification and development of the pars intercerebralis and pars lateralis, neuroendocrine command centers in the *Drosophila* brain. Developmental Biology.

[bib45] Domazet-Lošo T, Tautz D (2010). A phylogenetically based transcriptome age index mirrors ontogenetic divergence patterns. Nature.

[bib46] Domínguez-Cejudo MA, Casares F (2015). Anteroposterior patterning of *Drosophila* ocelli requires an anti-repressor mechanism within the hh pathway mediated by the Six3 gene Optix. Development.

[bib47] Dong Y, Friedrich M (2005). Comparative analysis of Wingless patterning in the embryonic grasshopper eye. Development Genes and Evolution.

[bib48] Dutta S, Dietrich J-E, Aspöck G, Burdine RD, Schier A, Westerfield M, Varga ZM (2005). pitx3 defines an equivalence domain for lens and anterior pituitary placode. Development.

[bib49] Economou AD, Telford MJ (2009). Comparative gene expression in the heads of *Drosophila melanogaster* and Tribolium castaneum and the segmental affinity of the *Drosophila* hypopharyngeal lobes. Evolution & Development.

[bib50] Erclik T, Hartenstein V, Lipshitz HD, McInnes RR (2008). Conserved role of the Vsx genes supports a monophyletic origin for bilaterian visual systems. Current Biology.

[bib51] Erclik T, Hartenstein V, McInnes RR, Lipshitz HD (2009). Eye evolution at high resolution: the neuron as a unit of homology. Developmental Biology.

[bib52] Eriksson BJ, Tait NN, Budd GE, Janssen R, Akam M (2010). Head patterning and Hox gene expression in an onychophoran and its implications for the arthropod head problem. Development Genes and Evolution.

[bib53] Erwin DH (2020). The origin of animal body plans: a view from fossil evidence and the regulatory genome. Development.

[bib54] Farnworth MS, Eckermann KN, Bucher G (2020). Sequence heterochrony led to A gain of functionality in an immature stage of the central complex: A fly-beetle insight. PLOS Biology.

[bib55] Farzana L, Brown SJ (2008). Hedgehog signaling pathway function conserved in Tribolium segmentation. Development Genes and Evolution.

[bib56] Fernández-Garre P, Rodríguez-Gallardo L, Gallego-Díaz V, Alvarez IS, Puelles L (2002). Fate map of the chicken neural plate at stage 4. Development.

[bib57] Finkelstein R, Smouse D, Capaci TM, Spradling AC, Perrimon N (1990). The orthodenticle gene encodes a novel homeo domain protein involved in the development of the *Drosophila* nervous system and ocellar visual structures. Genes & Development.

[bib58] Finkelstein R, Boncinelli E (1994). From fly head to mammalian forebrain: the story of otd and Otx. Trends in Genetics.

[bib59] Friedrich M, Singh A), Kango-Singh M (2013). Molecular Genetics of Axial Patterning, Growth and Disease in the Drosophila Eye.

[bib60] Friedrich M (2022). Coming into clear sight at last: Ancestral and derived events during chelicerate visual system development. BioEssays.

[bib61] Fritzenwanker JH, Gerhart J, Freeman RM, Lowe CJ (2014). The Fox/Forkhead transcription factor family of the hemichordate Saccoglossus kowalevskii. EvoDevo.

[bib62] Fu J, Posnien N, Bolognesi R, Fischer TD, Rayl P, Oberhofer G, Kitzmann P, Brown SJ, Bucher G (2012). Asymmetrically expressed axin required for anterior development in Tribolium. PNAS.

[bib63] Gans C, Northcutt RG (1983). Neural crest and the origin of vertebrates: A new head. Science.

[bib64] Gąsiorowski L, Børve A, Cherneva IA, Orús-Alcalde A, Hejnol A (2021). Molecular and morphological analysis of the developing nemertean brain indicates convergent evolution of complex brains in Spiralia. BMC Biology.

[bib65] Gehring WJ (1996). The master control gene for morphogenesis and evolution of the eye. Genes to Cells.

[bib66] Gehring WJ, Ikeo K (1999). Pax 6: mastering eye morphogenesis and eye evolution. Trends in Genetics.

[bib67] Gehring WJ (2005). New perspectives on eye development and the evolution of eyes and photoreceptors. The Journal of Heredity.

[bib68] Gestri G, Carl M, Appolloni I, Wilson SW, Barsacchi G, Andreazzoli M (2005). Six3 functions in anterior neural plate specification by promoting cell proliferation and inhibiting Bmp4 expression. Development.

[bib69] Ghanbari H, Seo HC, Fjose A, Brändli AW (2001). Molecular cloning and embryonic expression of *Xenopus* Six homeobox genes. Mechanisms of Development.

[bib70] Glenn Northcutt R (2005). The new head hypothesis revisited. Journal of Experimental Zoology. Part B, Molecular and Developmental Evolution.

[bib71] Grocott T, Tambalo M, Streit A (2012). The peripheral sensory nervous system in the vertebrate head: a gene regulatory perspective. Developmental Biology.

[bib72] Halder G, Callaerts P, Gehring WJ (1995). Induction of ectopic eyes by targeted expression of the eyeless gene in *Drosophila*. Science.

[bib73] Hartenstein V, Reh TA, Moses K (2002). Drosophila Eye Development.

[bib74] Hartenstein V (2006). The neuroendocrine system of invertebrates: a developmental and evolutionary perspective. The Journal of Endocrinology.

[bib75] Hartenstein V, Stollewerk A (2015). The evolution of early neurogenesis. Developmental Cell.

[bib76] He B, Buescher M, Farnworth MS, Strobl F, Stelzer EH, Koniszewski ND, Muehlen D, Bucher G (2019). An ancestral apical brain region contributes to the central complex under the control of *foxQ2* in the beetle *Tribolium*. eLife.

[bib77] Hejnol A, Lowe CJ (2015). Embracing the comparative approach: how robust phylogenies and broader developmental sampling impacts the understanding of nervous system evolution. Philosophical Transactions of the Royal Society of London. Series B, Biological Sciences.

[bib78] Hirth F, Kammermeier L, Frei E, Walldorf U, Noll M, Reichert H (2003). An urbilaterian origin of the tripartite brain: developmental genetic insights from *Drosophila*. Development.

[bib79] Hirth F (2010). On the origin and evolution of the tripartite brain. Brain, Behavior and Evolution.

[bib80] Holland PWH, Takahashi T (2005). The evolution of homeobox genes: Implications for the study of brain development. Brain Research Bulletin.

[bib81] Holland LZ, Short S (2008). Gene duplication, co-option and recruitment during the origin of the vertebrate brain from the invertebrate chordate brain. Brain, Behavior and Evolution.

[bib82] Hunnekuhl VS, Akam M (2014). An anterior medial cell population with an apical-organ-like transcriptional profile that pioneers the central nervous system in the centipede Strigamia maritima. Developmental Biology.

[bib83] Hunnekuhl VS, Akam M (2017). Formation and subdivision of the head field in the centipede *Strigamia maritima*, as revealed by the expression of head gap gene orthologues and *hedgehog* dynamics. EvoDevo.

[bib84] Inoue T, Nakamura S, Osumi N (2000). Fate mapping of the mouse prosencephalic neural plate. Developmental Biology.

[bib85] Ito K, Shinomiya K, Ito M, Armstrong JD, Boyan G, Hartenstein V, Harzsch S, Heisenberg M, Homberg U, Jenett A, Keshishian H, Restifo LL, Rössler W, Simpson JH, Strausfeld NJ, Strauss R, Vosshall LB, Insect Brain Name Working Group (2014). A systematic nomenclature for the insect brain. Neuron.

[bib86] Joly JS, Recher G, Brombin A, Ngo K, Hartenstein V (2016). A conserved developmental mechanism builds complex visual systems in insects and vertebrates. Current Biology.

[bib87] Kanayama M, Akiyama-Oda Y, Nishimura O, Tarui H, Agata K, Oda H (2011). Travelling and splitting of a wave of hedgehog expression involved in spider-head segmentation. Nature Communications.

[bib88] Kettle C, Johnstone J, Jowett T, Arthur H, Arthur W (2003). The pattern of segment formation, as revealed by engrailed expression, in a centipede with a variable number of segments. Evolution & Development.

[bib89] Kiecker C, Lumsden A (2005). Compartments and their boundaries in vertebrate brain development. Nature Reviews. Neuroscience.

[bib90] Kittelmann S, Ulrich J, Posnien N, Bucher G (2013). Changes in anterior head patterning underlie the evolution of long germ embryogenesis. Developmental Biology.

[bib91] Kitzmann P, Weißkopf M, Schacht MI, Bucher G (2017). foxQ2 has a key role in anterior head and central brain patterning in insects. Development.

[bib92] Klingler M, Bucher G (2022). The red flour beetle T. castaneum: elaborate genetic toolkit and unbiased large scale RNAi screening to study insect biology and evolution. EvoDevo.

[bib93] Koniszewski N (2011). Functional Analysis of Embryonic Brain Development in Tribolium Castaneum.

[bib94] Kunz T, Kraft KF, Technau GM, Urbach R (2012). Origin of *Drosophila* mushroom body neuroblasts and generation of divergent embryonic lineages. Development.

[bib95] Lacalli TC, Berrill NJ (1981). Structure and development of the apical organ in trochophores of *Spirobranchus polycerus, Phyllodoce maculata and Phyllodoce mucosa* (Polychaeta). Proceedings of the Royal Society of London. Series B. Biological Sciences.

[bib96] Lagutin OV, Zhu CC, Kobayashi D, Topczewski J, Shimamura K, Puelles L, Russell HRC, McKinnon PJ, Solnica-Krezel L, Oliver G (2003). Six3 repression of Wnt signaling in the anterior neuroectoderm is essential for vertebrate forebrain development. Genes & Development.

[bib97] Lan T, Zhao Y, Zhao F, He Y, Martinez P, Strausfeld NJ (2021). Leanchoiliidae reveals the ancestral organization of the stem euarthropod brain. Current Biology.

[bib98] Lavado A, Lagutin OV, Oliver G (2008). Six3 inactivation causes progressive caudalization and aberrant patterning of the mammalian diencephalon. Development.

[bib99] Leclère L, Bause M, Sinigaglia C, Steger J, Rentzsch F (2016). Development of the aboral domain in Nematostella requires β-catenin and the opposing activities of Six3/6 and Frizzled5/8. Development.

[bib100] Leite DJ, Schönauer A, Blakeley G, Harper A, Garcia-Castro H, Baudouin-Gonzalez L, Wang R, Sarkis N, Nikola AG, Koka VSP, Kenny NJ, Turetzek N, Pechmann M, Solana J, McGregor AP (2022). An atlas of spider development at single-cell resolution provides new insights into arthropod embryogenesis. bioRxiv.

[bib101] Leuzinger S, Hirth F, Gerlich D, Acampora D, Simeone A, Gehring WJ, Finkelstein R, Furukubo-Tokunaga K, Reichert H (1998). Equivalence of the fly orthodenticle gene and the human OTX genes in embryonic brain development of *Drosophila*. Development.

[bib102] Lev O, Chipman AD (2021). Development of the Pre-gnathal Segments in the Milkweed Bug *Oncopeltus fasciatus* Suggests They Are Not Serial Homologs of Trunk Segments. Frontiers in Cell and Developmental Biology.

[bib103] Lev O, Edgecombe GD, Chipman AD (2022). Serial homology and segment identity in the arthropod head. Integrative Organismal Biology.

[bib104] Li HS, Yang JM, Jacobson RD, Pasko D, Sundin O (1994). Pax-6 is first expressed in a region of ectoderm anterior to the early neural plate: implications for stepwise determination of the lens. Developmental Biology.

[bib105] Li Y, Brown SJ, Hausdorf B, Tautz D, Denell RE, Finkelstein R (1996). Two orthodenticle-related genes in the short-germ beetle Tribolium castaneum. Development Genes and Evolution.

[bib106] Lichtneckert R, Reichert H (2005). Insights into the urbilaterian brain: conserved genetic patterning mechanisms in insect and vertebrate brain development. Heredity.

[bib107] Lichtneckert R, Nobs L, Reichert H (2008). Empty spiracles is required for the development of olfactory projection neuron circuitry in *Drosophila*. Development.

[bib108] Litsiou A, Hanson S, Streit A (2005). A balance of FGF, BMP and WNT signalling positions the future placode territory in the head. Development.

[bib109] Liu Z, Yang X, Dong Y, Friedrich M (2006). Tracking down the “head blob”: comparative analysis of wingless expression in the developing insect procephalon reveals progressive reduction of embryonic visual system patterning in higher insects. Arthropod Structure & Development.

[bib110] Loesel R, Nässel DR, Strausfeld NJ (2002). Common design in a unique midline neuropil in the brains of arthropods. Arthropod Structure & Development.

[bib111] Lowe CJ, Wu M, Salic A, Evans L, Lander E, Stange-Thomann N, Gruber CE, Gerhart J, Kirschner M (2003). Anteroposterior patterning in hemichordates and the origins of the chordate nervous system. Cell.

[bib112] Lowe CJ (2008). Molecular genetic insights into deuterostome evolution from the direct-developing hemichordate Saccoglossus kowalevskii. Philosophical Transactions of the Royal Society of London. Series B, Biological Sciences.

[bib113] Mallamaci A, Iannone R, Briata P, Pintonello L, Mercurio S, Boncinelli E, Corte G (1998). EMX2 protein in the developing mouse brain and olfactory area. Mechanisms of Development.

[bib114] Marlow H, Tosches MA, Tomer R, Steinmetz PR, Lauri A, Larsson T, Arendt D (2014). Larval body patterning and apical organs are conserved in animal evolution. BMC Biology.

[bib115] Martín-Durán JM, Wolff GH, Strausfeld NJ, Hejnol A (2016). The larval nervous system of the penis worm Priapulus caudatus (Ecdysozoa). Philosophical Transactions of the Royal Society of London. Series B, Biological Sciences.

[bib116] Martín-Durán JM, Hejnol A (2021). A developmental perspective on the evolution of the nervous system. Developmental Biology.

[bib117] Martinez-Arias A, Lawrence PA (1985). Parasegments and compartments in the *Drosophila* embryo. Nature.

[bib118] Meyer NP, Carrillo-Baltodano A, Moore RE, Seaver EC (2015). Nervous system development in lecithotrophic larval and juvenile stages of the annelid Capitella teleta. Frontiers in Zoology.

[bib119] Mizunami M (1995a). Functional diversity of neural organization in insect ocellar systems. Vision Research.

[bib120] Mizunami M (1995b). Neural organization of ocellar pathways in the cockroach brain. The Journal of Comparative Neurology.

[bib121] Momose T, Derelle R, Houliston E (2008). A maternally localised Wnt ligand required for axial patterning in the cnidarian Clytia hemisphaerica. Development.

[bib122] Moreno N, Bachy I, Rétaux S, González A (2003). Pallial origin of mitral cells in the olfactory bulbs of *Xenopus*. Neuroreport.

[bib123] Nagy LM, Carroll S (1994). Conservation of wingless patterning functions in the short-germ embryos of Tribolium castaneum. Nature.

[bib124] Nie W, Stronach B, Panganiban G, Shippy T, Brown S, Denell R (2001). Molecular characterization of Tclabial and the 3’ end of the Tribolium homeotic complex. Development Genes and Evolution.

[bib125] Nieber F, Pieler T, Henningfeld KA (2009). Comparative expression analysis of the neurogenins in *Xenopus tropicalis* and *Xenopus laevis*. Developmental Dynamics.

[bib126] Nielsen C (1999). Origin of the chordate central nervous system - and the origin of chordates. Development Genes and Evolution.

[bib127] Nielsen C, Martinez P (2003). Patterns of gene expression: homology or homocracy?. Development Genes and Evolution.

[bib128] Nielsen C (2005). Larval and adult brains. Evolution & Development.

[bib129] Nilsson DE, Arendt D (2008). Eye evolution: the blurry beginning. Current Biology.

[bib130] Ntini E, Wimmer EA (2011a). Unique establishment of procephalic head segments is supported by the identification of cis-regulatory elements driving segment-specific segment polarity gene expression in *Drosophila*. Development Genes and Evolution.

[bib131] Ntini E, Wimmer EA (2011b). Second order regulator Collier directly controls intercalary-specific segment polarity gene expression. Developmental Biology.

[bib132] Oberhofer G, Grossmann D, Siemanowski JL, Beissbarth T, Bucher G (2014). Wnt/β-catenin signaling integrates patterning and metabolism of the insect growth zone. Development.

[bib133] Ogawa Y, Shiraki T, Fukada Y, Kojima D (2021). Foxq2 determines blue cone identity in zebrafish. Science Advances.

[bib134] Oliver G, Mailhos A, Wehr R, Copeland NG, Jenkins NA, Gruss P (1995). Six3, a murine homologue of the sine oculis gene, demarcates the most anterior border of the developing neural plate and is expressed during eye development. Development.

[bib135] Omoto JJ, Nguyen BCM, Kandimalla P, Lovick JK, Donlea JM, Hartenstein V (2018). Neuronal Constituents and Putative Interactions Within the *Drosophila* Ellipsoid Body Neuropil. Frontiers in Neural Circuits.

[bib136] Ortega-Hernández J, Janssen R, Budd GE (2017). Origin and evolution of the panarthropod head - A palaeobiological and developmental perspective. Arthropod Structure & Development.

[bib137] Pandur PD, Moody SA (2000). *Xenopus* Six1 gene is expressed in neurogenic cranial placodes and maintained in the differentiating lateral lines. Mechanisms of Development.

[bib138] Pani AM, Mullarkey EE, Aronowicz J, Assimacopoulos S, Grove EA, Lowe CJ (2012). Ancient deuterostome origins of vertebrate brain signalling centres. Nature.

[bib139] Patel NH, Kornberg TB, Goodman CS (1989). Expression of engrailed during segmentation in grasshopper and crayfish. Development.

[bib140] Pfeiffer K, Homberg U (2014). Organization and functional roles of the central complex in the insect brain. Annual Review of Entomology.

[bib141] Pommereit D, Pieler T, Hollemann T (2001). Xpitx3: a member of the Rieg/Pitx gene family expressed during pituitary and lens formation in *Xenopus laevis*. Mechanisms of Development.

[bib142] Posnien N, Bucher G (2010a). Formation of the insect head involves lateral contribution of the intercalary segment, which depends on Tc-labial function. Developmental Biology.

[bib143] Posnien N, Schinko JB, Kittelmann S, Bucher G (2010b). Genetics, development and composition of the insect head--A beetle’s view. Arthropod Structure & Development.

[bib144] Posnien N, Koniszewski N, Bucher G (2011a). Insect Tc-six4 marks a unit with similarity to vertebrate placodes. Developmental Biology.

[bib145] Posnien N, Koniszewski NDB, Hein HJ, Bucher G (2011b). Candidate gene screen in the red flour beetle Tribolium reveals six3 as ancient regulator of anterior median head and central complex development. PLOS Genetics.

[bib146] Puelles L, Fernández-Garre P, Sánchez-Arrones L, García-Calero E, Rodríguez-Gallardo L (2005). Correlation of a chicken stage 4 neural plate fate map with early gene expression patterns. Brain Research. Brain Research Reviews.

[bib147] Range RC, Wei Z (2016). An anterior signaling center patterns and sizes the anterior neuroectoderm of the sea urchin embryo. Development.

[bib148] Reichert H, Simeone A (1999). Conserved usage of gap and homeotic genes in patterning the CNS. Current Opinion in Neurobiology.

[bib149] Reichert H (2005). A tripartite organization of the urbilaterian brain: developmental genetic evidence from *Drosophila*. Brain Research Bulletin.

[bib150] Remane A (1950). Enstehung der Metamerie der Wirbellosen. Zoologischer Anzeiger.

[bib151] Rempel GJ (1975). The evolution of the insect head: the endless dispute. Quaestiones Entomologicae.

[bib152] Rhinn M, Lun K, Luz M, Werner M, Brand M (2005). Positioning of the midbrain-hindbrain boundary organizer through global posteriorization of the neuroectoderm mediated by Wnt8 signaling. Development.

[bib153] Rhinn M, Picker A, Brand M (2006). Global and local mechanisms of forebrain and midbrain patterning. Current Opinion in Neurobiology.

[bib154] Rogers BT, Kaufman TC (1997). Structure of the insect head in ontogeny and phylogeny: a view from *Drosophila*. International Review of Cytology.

[bib155] Rubenstein JL, Shimamura K, Martinez S, Puelles L (1998). Regionalization of the prosencephalic neural plate. Annual Review of Neuroscience.

[bib156] Samadi L, Schmid A, Eriksson BJ (2015). Differential expression of retinal determination genes in the principal and secondary eyes of Cupiennius salei Keyserling (1877). EvoDevo.

[bib157] Schaeper ND, Pechmann M, Damen WGM, Prpic N-M, Wimmer EA (2010). Evolutionary plasticity of collier function in head development of diverse arthropods. Developmental Biology.

[bib158] Schinko JB, Kreuzer N, Offen N, Posnien N, Wimmer EA, Bucher G (2008). Divergent functions of orthodenticle, empty spiracles and buttonhead in early head patterning of the beetle Tribolium castaneum (Coleoptera). Developmental Biology.

[bib159] Schlosser G, Ahrens K (2004). Molecular anatomy of placode development in *Xenopus laevis*. Developmental Biology.

[bib160] Schlosser G (2005). Evolutionary origins of vertebrate placodes: insights from developmental studies and from comparisons with other deuterostomes. Journal of Experimental Zoology. Part B, Molecular and Developmental Evolution.

[bib161] Schlosser G (2010). Making senses development of vertebrate cranial placodes. International Review of Cell and Molecular Biology.

[bib162] Schlosser G (2014). Early embryonic specification of vertebrate cranial placodes. Wiley Interdisciplinary Reviews. Developmental Biology.

[bib163] Schlosser G (2015). Vertebrate cranial placodes as evolutionary innovations--the ancestor’s tale. Current Topics in Developmental Biology.

[bib164] Schlosser G (2017). From so simple a beginning - what amphioxus can teach us about placode evolution. The International Journal of Developmental Biology.

[bib165] Schmidt-Ott U, Technau GM (1992). Expression of en and wg in the embryonic head and brain of *Drosophila* indicates a refolded band of seven segment remnants. Development.

[bib166] Schoenemann B, Clarkson ENK (2023). The median eyes of trilobites. Scientific Reports.

[bib167] Scholtz G, Patel NH, Dohle W (1994). Serially homologous engrailed stripes are generated via different cell lineages in the germ band of amphipod crustaceans (Malacostraca, Peracarida). The International Journal of Developmental Biology.

[bib168] Scholtz G, Edgecombe GD (2006). The evolution of arthropod heads: reconciling morphological, developmental and palaeontological evidence. Development Genes and Evolution.

[bib169] Schomburg C, Turetzek N, Schacht MI, Schneider J, Kirfel P, Prpic NM, Posnien N (2015). Molecular characterization and embryonic origin of the eyes in the common house spider Parasteatoda tepidariorum. EvoDevo.

[bib170] Schroder R, Eckert C, Wolff C, Tautz D (2000). Conserved and divergent aspects of terminal patterning in the beetle Tribolium castaneum. PNAS.

[bib171] Sen S, Reichert H, VijayRaghavan K (2013). Conserved roles of ems/Emx and otd/Otx genes in olfactory and visual system development in *Drosophila* and mouse. Open Biology.

[bib172] Shimamura K, Hartigan DJ, Martinez S, Puelles L, Rubenstein JL (1995). Longitudinal organization of the anterior neural plate and neural tube. Development.

[bib173] Shinozaki K, Miyagi T, Yoshida M, Miyata T, Ogawa M, Aizawa S, Suda Y (2002). Absence of Cajal-Retzius cells and subplate neurons associated with defects of tangential cell migration from ganglionic eminence in Emx1/2 double mutant cerebral cortex. Development.

[bib174] Shubin N, Tabin C, Carroll S (1997). Fossils, genes and the evolution of animal limbs. Nature.

[bib175] Shubin N, Tabin C, Carroll S (2009). Deep homology and the origins of evolutionary novelty. Nature.

[bib176] Siemanowski J, Richter T, Dao VA, Bucher G (2015). Notch signaling induces cell proliferation in the labrum in a regulatory network different from the thoracic legs. Developmental Biology.

[bib177] Siewing R (1963). Zum Problem der Arthropodenkopfsegmentierung. Zoologischer Anzeiger.

[bib178] Simeone A, Acampora D, Gulisano M, Stornaiuolo A, Boncinelli E (1992a). Nested expression domains of four homeobox genes in developing rostral brain. Nature.

[bib179] Simeone A, Gulisano M, Acampora D, Stornaiuolo A, Rambaldi M, Boncinelli E (1992b). Two vertebrate homeobox genes related to the *Drosophila* empty spiracles gene are expressed in the embryonic cerebral cortex. The EMBO Journal.

[bib180] Sinigaglia C, Busengdal H, Leclère L, Technau U, Rentzsch F (2013). The bilaterian head patterning gene six3/6 controls aboral domain development in a cnidarian. PLOS Biology.

[bib181] Sintoni S, Fabritius-Vilpoux K, Harzsch S (2007). The Engrailed-expressing secondary head spots in the embryonic crayfish brain: examples for a group of homologous neurons in Crustacea and Hexapoda?. Development Genes and Evolution.

[bib182] Skeath JB, Thor S (2003). Genetic control of *Drosophila* nerve cord development. Current Opinion in Neurobiology.

[bib183] Slack JMW, Holland PWH, Graham CF (1993). The zootype and the phylotypic stage. Nature.

[bib184] Smith FW, Cumming M, Goldstein B (2018). Analyses of nervous system patterning genes in the tardigrade *Hypsibius exemplaris* illuminate the evolution of panarthropod brains. EvoDevo.

[bib185] Snodgrass RE (1935). Principles of Insect Morphology.

[bib186] Snodgrass RE (1953). The metamorphosis of a fly’s head. Smithsonian Miscellaneous Collections.

[bib187] Stahi R, Chipman AD (2016). Blastoderm segmentation in *Oncopeltus fasciatus* and the evolution of insect segmentation mechanisms. Proceedings. Biological Sciences.

[bib188] Steinmetz PR, Urbach R, Posnien N, Eriksson J, Kostyuchenko RP, Brena C, Guy K, Akam M, Bucher G, Arendt D (2010). Six3 demarcates the anterior-most developing brain region in bilaterian animals. EvoDevo.

[bib189] Steinmetz PRH, Kostyuchenko RP, Fischer A, Arendt D (2011). The segmental pattern of otx, gbx, and Hox genes in the annelid *Platynereis dumerilii*. Evolution & Development.

[bib190] Strausfeld NJ (1976). Atlas of an Insect Brain.

[bib191] Strausfeld NJ (2012). Arthropod Brains: Evolution, Functional Elegance, and Historical Significance.

[bib192] Strausfeld NJ, Hirth F (2013). Deep homology of arthropod central complex and vertebrate basal ganglia. Science.

[bib193] Strausfeld NJ, Hou X, Sayre ME, Hirth F (2022). The lower Cambrian lobopodian *Cardiodictyon* resolves the origin of euarthropod brains. Science.

[bib194] Streit A (2007). The preplacodal region: an ectodermal domain with multipotential progenitors that contribute to sense organs and cranial sensory ganglia. The International Journal of Developmental Biology.

[bib195] Stuart JJ, Brown SJ, Beeman RW, Denell RE (1991). A deficiency of the homeotic complex of the beetle Tribolium. Nature.

[bib196] Taira M, Hayes WP, Otani H, Dawid IB (1993). Expression of LIM class homeobox gene Xlim-3 in *Xenopus* development is limited to neural and neuroendocrine tissues. Developmental Biology.

[bib197] Tautz D, Friedrich M, Schröder R (1994). Insect embryogenesis - what is ancestral and what is derived?. Development.

[bib198] Technau GM, Berger C, Urbach R (2006). Generation of cell diversity and segmental pattern in the embryonic central nervous system of *Drosophila*. Developmental Dynamics.

[bib199] Telford MJ, Thomas RH (1998). Expression of homeobox genes shows chelicerate arthropods retain their deutocerebral segment. PNAS.

[bib200] Tessmar-Raible K (2007). The evolution of neurosecretory centers in bilaterian forebrains: insights from protostomes. Seminars in Cell & Developmental Biology.

[bib201] Tessmar-Raible K, Raible F, Christodoulou F, Guy K, Rembold M, Hausen H, Arendt D (2007). Conserved sensory-neurosecretory cell types in annelid and fish forebrain: insights into hypothalamus evolution. Cell.

[bib202] Tomer R, Denes AS, Tessmar-Raible K, Arendt D (2010). Profiling by image registration reveals common origin of annelid mushroom bodies and vertebrate pallium. Cell.

[bib203] Toresson H, Martinez-Barbera JP, Bardsley A, Caubit X, Krauss S (1998). Conservation of BF-1 expression in amphioxus and zebrafish suggests evolutionary ancestry of anterior cell types that contribute to the vertebrate telencephalon. Development Genes and Evolution.

[bib204] Tremblay JJ, Lanctôt C, Drouin J (1998). The pan-pituitary activator of transcription, Ptx1 (pituitary homeobox 1), acts in synergy with SF-1 and Pit1 and is an upstream regulator of the Lim-homeodomain gene Lim3/Lhx3. Molecular Endocrinology.

[bib205] Urbach R, Technau GM (2003a). Molecular markers for identified neuroblasts in the developing brain of *Drosophila*. Development.

[bib206] Urbach R, Technau GM (2003b). Segment polarity and DV patterning gene expression reveals segmental organization of the *Drosophila* brain. Development.

[bib207] Urbach R, Technau GM (2003c). Early steps in building the insect brain: neuroblast formation and segmental patterning in the developing brain of different insect species. Arthropod Structure & Development.

[bib208] Urbach R (2007). A procephalic territory in *Drosophila* exhibiting similarities and dissimilarities compared to the vertebrate midbrain/hindbrain boundary region. Neural Development.

[bib209] Urbach R, Jussen D, Technau GM (2016). Gene expression profiles uncover individual identities of gnathal neuroblasts and serial homologies in the embryonic CNS of *Drosophila*. Development.

[bib210] Vopalensky P, Kozmik Z (2009). Eye evolution: common use and independent recruitment of genetic components. Philosophical Transactions of the Royal Society of London. Series B, Biological Sciences.

[bib211] Wagner GP (2007). The developmental genetics of homology. Nature Reviews. Genetics.

[bib212] Walsh KT, Doe CQ (2017). *Drosophila* embryonic type II neuroblasts: origin, temporal patterning, and contribution to the adult central complex. Development.

[bib213] Weber H (1966). Grundriss Der Insektenkunde.

[bib214] Wei Z, Yaguchi J, Yaguchi S, Angerer RC, Angerer LM (2009). The sea urchin animal pole domain is a Six3-dependent neurogenic patterning center. Development.

[bib215] Westheide W, Rieger R (1996). Spezielle Zoologie; Teil 1: Einzeller Und Wirbellose Tiere.

[bib216] Wheeler SR, Carrico ML, Wilson BA, Brown SJ, Skeath JB (2003). The expression and function of the achaete-scute genes in Tribolium castaneum reveals conservation and variation in neural pattern formation and cell fate specification. Development.

[bib217] Wheeler SR, Carrico ML, Wilson BA, Skeath JB (2005). The Tribolium columnar genes reveal conservation and plasticity in neural precursor patterning along the embryonic dorsal–ventral axis. Developmental Biology.

[bib218] Wollesen T, Rodríguez Monje SV, Todt C, Degnan BM, Wanninger A (2015). Ancestral role of Pax2/5/8 in molluscan brain and multimodal sensory system development. BMC Evolutionary Biology.

[bib219] Yaguchi S, Yaguchi J, Angerer RC, Angerer LM (2008). A Wnt-FoxQ2-Nodal Pathway Links Primary and Secondary Axis Specification in Sea Urchin Embryos. Developmental Cell.

[bib220] Yaguchi J, Takeda N, Inaba K, Yaguchi S (2016). Cooperative Wnt-Nodal Signals Regulate the Patterning of Anterior Neuroectoderm. PLOS Genetics.

[bib221] Yamada G, Ueno K, Nakamura S, Hanamure Y, Yasui K, Uemura M, Eizuru Y, Mansouri A, Blum M, Sugimura K (1997). Nasal and Pharyngeal Abnormalities Caused by the Mouse Goosecoid Gene Mutation. Biochemical and Biophysical Research Communications.

[bib222] Yang X, Weber M, Zarinkamar N, Posnien N, Friedrich F, Wigand B, Beutel R, Damen WGM, Bucher G, Klingler M, Friedrich M (2009a). Probing the *Drosophila* retinal determination gene network in Tribolium (II): The Pax6 genes eyeless and twin of eyeless. Developmental Biology.

[bib223] Yang X, ZarinKamar N, Bao R, Friedrich M (2009b). Probing the *Drosophila* retinal determination gene network in Tribolium (I): The early retinal genes dachshund, eyes absent and sine oculis. Developmental Biology.

[bib224] Yu JK, Holland ND, Holland LZ (2003). AmphiFoxQ2, a novel winged helix/forkhead gene, exclusively marks the anterior end of the amphioxus embryo. Development Genes and Evolution.

