# Supplementary material for: Gene expression mapping of the neuroectoderm across phyla – conservation and divergence of early brain anlagen between insects and vertebrates
Source: eLife. 2023 Sep 26;12:e92242. doi: 10.7554/eLife.92242 (PMC10522337; doi:10.7554/eLife.92242)
Supplement: Supplementary file 1. [file elife-92242-supp1.pdf]

Supplementary file 1

Contents

Why do fly data lead to different conclusions with respect to the mapping of the IHB? 1
Ganglia are segmental structures – not parasegmental 2
The diverging location of eye anlagen in flies versus beetles 2
Notes on expression patterns shown in Fig. 1 3
Notes on expression patterns shown in Fig. 2 3
Notes on expression patterns and fate maps shown in Fig. 3 4
References 4

Why do fly data lead to different conclusions with respect to the mapping of the IHB?

Three issues may explain the divergent interpretations in flies. First, Hirth et al. used a more advanced stage (stage 13/14) compared to Urbach (stage 11). Hence, the former data are based on expression within an already visible developing CNS while the latter is based on the late neuroectoderm where neuroblasts just start to delaminate. Hence, morphogenetic movements and/or secondary functions of these genes may affect expression of the later stage. Second, he used a small number of genes such that potential idiosyncrasies of the expression of single genes would not be balanced by other data. Indeed, we find that different markers hint to slightly different boundaries (red broken lines in Fig. 2A). Third, a key argument for positioning the MHB at the antennal/intercalary boundary was the expression of *shaven/Pax2* and PoxN, which were interpreted to be a Pax2/5/8 orthologs. Specifically, Hirth et al. mapped the earliest *shaven/Pax2* expression to the prospective deuto-tritocerebral boundary (i.e. boundary between antennal and intercalary segments) in stage 9 embryos (Fig 2A in that paper). Taking into account the careful re-mapping of *wg* and *hh* patterns in the procephalic ectoderm (Ntini and Wimmer, 2011) (see schematic in Fig. 1B-D and hh-patterns in Fig. 4G in that paper), the *shaven/Pax2* expression shown by Hirth et al. could be mapped to the deutocerebral-protocerebral boundary as well . However, double stainings would be required to confidently resolve that matter. For later stages, Hirth et al. use an enhancer trap line to visualize *shaven/Pax2* expression and found segmentally reiterated patterns in addition to signals in the deutocerebrum and the protocerebrum. Co-expression with PoxN (assumed to be another Pax2/5/8 ortholog) was found in the deutocerebrum but not the other domains. This was used as argument to use the Pax2 domain at the deutocerebral-tritocerebral boundary (but none of the other domains) to map the MHB-homolog (Hirth et al., 2003). In contrast, Urbach used mRNA stainings of *shaven/Pax2* and reported a more refined pattern compared to the enhancer trap. He found no *shaven/Pax2* expression in the neuroectoderm nor in any brain neuroblast. He came to the conclusion that the later segmental *shaven/Pax2* activity was too late for patterning the neuroectoderm. (Urbach, 2007).

Both studies interpreted PoxN as a second Pax2/5/8 ortholog. However, phylogenetic investigations based on the expanded genome sequences available nowadays indicate that this gene is actually not orthologous to Pax2/5/8 (Smith et al., 2018; Wollesen et al., 2015).

Ganglia are segmental structures – not parasegmental

In our scheme, the ganglia reflect the segmental frame as opposed to the embryonic parasegmental frame that was suggested by others (Deutsch, 2004). This claim is based on the following reasons: Engrailed and hedgehog mark the future posterior part of each segment but the anterior part of each parasegment (broken black lines shown only for the posterior segments in Fig. 2) (Martinez-Arias and Lawrence, 1985). Hence, engrailed expression and function is a perfect marker to distinguish between these two hypotheses. If ganglia are segmental, engrailed is expected at their posterior part while it should be found at the anterior of each ganglion if they were parasegmental structures.

In Drosophila and Schistocerca, engrailed positive neural cell bodies are located in the posterior of each neuromere (Kumar et al., 2009; Siegler and Jia, 1999; Siegler and Pankhaniya, 1997). In Drosophila, engrailed positive neurons project through the posterior commissure and in engrailed mutants, the posterior commissure is affected or even absent (Joly et al., 2007). Further, the posterior row of neuroblasts of each hemineuromere is engrailed positive in Drosophila (Urbach and Technau, 2003a) and a malocostracan crustacean (Ungerer and Scholtz, 2008). Intriguingly, a median engrailed positive neuroblast gives rise to probably homologous neurons in flies and malacostracan crustaceans, which have their cell bodies directly posterior-adjacent to the posterior commissure and project through this commissure and the posterior segmental nerve (Technau et al., 2006; Ungerer and Scholtz, 2008). Taken together, all these data are strong evidence for a contribution of engrailed positive cells to the posterior parts of the ganglia, strongly favoring the hypothesis of ganglia being segmental structures.

The diverging location of eye anlagen in flies versus beetles

The co-expression of the transcription factors *sine oculis* and *eyes absent* in Drosophila embryos marks the anlagen of the eye field (including the anlagen of the compound eyes) (Chang et al., 2001; Friedrich, 2013). For this discussion we use *sine oculis* as a very faithful marker for the eye field because a respective imaging line in flies marked eye neuroblasts, eye target neurons of the larval eye and the some optic lobe pioneers among other cells (Chang et al., 2003). In fly embryos, *sine oculis* starts expression in a stripe across the dorsal midline of the embryonic head before it splits into the two lateral eye anlagen. Hence, the early eye anlagen were mapped to a continuous dorsal eye field, which later separates into two lateral eye anlagen, which migrate ventrally (Chang et al., 2001; Friedrich, 2013). This seems not unlike the situation in vertebrates where the two eye anlagen are located in close proximity at the midline.

The situation in beetles is different. Here, *sine oculis* starts being expressed in two separate domains, which are very actually very far apart from each other (Fig. 1 F) (Posnien et al., 2011a). In line with this finding, the eye anlagen of beetles and grasshoppers have been mapped to these separate regions based on wingless and eyeless expression (Dong and Friedrich, 2005; Liu et al., 2006; Yang et al., 2009a; Yang et al., 2009b).

Which of these insect species should be used for the comparison with vertebrates? Within insects, the head anlagen typically emerge at a *ventral median* or even posterior position in the blastoderm and they start out in an essentially two dimensional setting. Indeed, the expression of all genes shown in Fig. 1 start out in this 2D setting, where the eye anlagen emerge on opposite sides of the head lobes (see eye anlagen marked in Fig. 1F). Only later, the 2D anlagen undergo morphogenesis to fold into a 3 dimensional head according to the "bend and zipper" model of head formation (Posnien and Bucher, 2010; Posnien et al., 2010). In flies, in contrast, the head anlagen are located at an *anterior dorsal* position (Posnien et al., 2010). When considering the different morphogenetic movements of the head anlagen, the later aspects of expression accord quite well between these insects. Actually, when dissecting the fly germband from the egg and flattening it like Urbach et al. has been doing (e.g. Technau et al., 2006; Urbach and Technau, 2003b), the two species's early head anlagen look quite similar.

In summary, only the situation at early embryonic stages strongly diverges such that early patterning events are likely to be under the control of different signaling cues (e.g. the dorsal versus ventral signals) and it is likely that the early head patterning system of Drosophila was modified to this new environment. For example, mutating dpp as the key dorsal morphogen leads to an enlargement of the head anlagen in beetles (both morphologically and in terms of otd expression) (van der Zee et al., 2006) similar to the effect in vertebrates (Reversade et al., 2005). However, at least on the level of otd expression no effect was not found in fly dpp mutants (van der Zee et al., 2006).

Hence, we assign the differences of the early eye field specification observed between flies and beetles to the different morphological settings at early stages and we have argued previously that the beetle situation is probably more typical for insects (Posnien et al., 2010). Importantly, the Drosophila situation resembles the beetle situation at later stages of embryogenesis and the conclusions drawn from marker gene expression in beetles is mostly reflected by fly data as well (e.g. compare the data shown in Urbach 2007 with our data).

Notes on expression patterns shown in Fig. 1

The expression patterns for the vertebrate genes were extracted from late gastrulation and early neurulation stages of different vertebrates: Embryonic day 7-8.5 (mouse), 8-12 hours post fertilization (zebrafish), stage 11-14 (Xenopus laevis) and HH4-7.5 (chick) (see Table S1 in Posnien 2011 for references). They were plotted onto the early 4-6 somite stage mouse neural plate (Rubenstein, 1997).

The Tribolium fgf8 and Dll expression is based on Beermann and Schröder, 2008 and Beerman et al. 2001, respectively. Engrailed expression is based on Brown et al., 1994. Pictures of stained embryos of the respective stages were manually transformed to fit a standard embryo using Tc-wg co-expression and morphology of the head lobes as criteria (Adobe Illustrator CS4 software). The outline of the expression domains were redrawn (Adobe Illustrator CS4 software). This allowed mapping the relative position of all combinations of expression patterns in “virtual double stainings” but the exact patterns of co-expression remain to be elucidated by direct double-stainings.

Notes on expression patterns shown in Fig. 2

FGF-8 is expressed in the antennal segment of Tribolium but not Drosophila (Beermann and Schroder, 2008; Posnien et al., 2011b; Urbach, 2007). Its posterior boundary coincides with the one from wingless (Posnien et al., 2011b) while the anterior boundary is less well mapped but might be at some distance to the ocular hh-stripe. Other aspects of FGF-8 expression are omitted. Otd has a very narrow overlap with ems expression (not shown) (Schinko et al., 2008), while the anterior boundary of ems is exactly abutting the ocular wg stripe (unpublished data). Therefore, we show otd expression extending a bit into the hh-domain but not into the antennal segment. Note that in flies, otd seems to mark parts of the deutocerebrum/antennal segment (Hirth et al., 2003; Urbach, 2007). No ungp/gbx data was available for Tribolium but based on data from flies and other animals it is likely to abut the otd expression (not depicted in Fig. 2). Labial/Hox1 is the anterior-most expressed Hox gene in insects and many other animals but in the vertebrate CNS, it is expressed subterminally in rhombomere 4 (taken from Urbach 2007). Hence, the anterior boundary of HOX marked tissue is defined by non-orthologous Hox genes. Engrailed forms a stripe at the vertebrate MHB but in all insect segment boundaries except for the ocular/antennal boundary. Here, engrailed expression is reduced to a domain called the “head spot”, which we assume to be a deviation from the ancestral stripe like pattern. In line with this, hh is expressed in a stripe like pattern at the ocular/antennal parasegment boundary and the respective engrailed domain is indeed a stripe in spiders and a crustacean (Damen, 2002; Patel et al., 1989). The btd domains anterior to the antennal segment emerge later in development and do not have the stripe-like appearance of the posterior segments (Schinko et al., 2008). One pre-antennal domain seems to be located in the six3 positive tissue and one behind it. However, their positions have not been mapped precisely.

Notes on expression patterns and fate maps shown in Fig. 3

The mouse fate map and expression of otx, emx and pax6 is based on (Inoue et al., 2000; Shimamura et al., 1995). Six3 expression was added based on the description in (Oliver et al., 1995) considering co-expression with both otx and pax6 and its expression in optic vesicle anlagen. The anlagen of the olfactory bulb was added based on a chick fatemap shown in (Cobos et al., 2001) and considering that six3 expression in olfactory bulb anlagen was not stated in mouse (Oliver et al., 1995) and seems to be median to those anlagen in chick as well (Puelles et al., 2005). The expression of the panplacodal marker six4 and the location of the placodes is based on *Xenopus* data summarized in (Schlosser, 2014). The expression patterns of the insect fate-maps are based on Figure 1 and data from (Posnien et al., 2011c). The location of the larval/compound eye anlagen betweenm the two lateral wingless domains is based on (Luan et al., 2014; Yang et al., 2009a). The ocelli anlagen are placed within the six4/eya marked tissue, because they have been mapped to a respective eya expression in grasshopper. Further, we tentatively placed the anterior ocelli within six3 and otd positive regions while the posterior ocelli were postulated to be six3 negative and maybe rx positive.

References

Beermann, A. and Schroder, R. (2008). Sites of Fgf signalling and perception during embryogenesis of the beetle Tribolium castaneum. *Development genes and evolution* **218**, 153–67.

Chang, T., Mazotta, J., Dumstrei, K., Dumitrescu, A. and Hartenstein, V. (2001). Dpp and Hh signaling in the Drosophila embryonic eye field. *Development* **128**, 4691–704.

Chang, T., Younossi-Hartenstein, A. and Hartenstein, V. (2003). Development of neural lineages derived from the sine oculis positive eye field of Drosophila. *Arthropod Struct Dev* **32**, 303–317.

Cobos, I., Shimamura, K., Rubenstein, J. L., Martínez, S. and Puelles, L. (2001). Fate map of the avian anterior forebrain at the four-somite stage, based on the analysis of quail-chick chimeras. *Dev Biol* **239**, 46–67.

Damen, W. G. M. (2002). Parasegmental organization of the spider embryo implies that the parasegment is an evolutionary conserved entity in arthropod embryogenesis. *Development* **129**, 1239–1250.

Deutsch, J. S. (2004). Segments and parasegments in arthropods: a functional perspective. *Bioessays* **26**, 1117–1125.

Dong, Y. and Friedrich, M. (2005). Comparative analysis of Wingless patterning in the embryonic grasshopper eye. *Dev Genes Evol* **215**, 177–97.

Friedrich, M. (2013). Development and Evolution of the Drosophila Bolwig's Organ: A Compound Eye Relict. In *Molecular Genetics of Axial Patterning, Growth and Disease in the Drosophila Eye* (ed. Singh, A.) and Kango-Singh, M.), pp. 329–357. New York, NY: Springer.

Hirth, F., Kammermeier, L., Frei, E., Walldorf, U., Noll, M. and Reichert, H. (2003). An urbilaterian origin of the tripartite brain: developmental genetic insights from Drosophila. *Development* **130**, 2365–73.

Inoue, T., Nakamura, S. and Osumi, N. (2000). Fate mapping of the mouse prosencephalic neural plate. *Dev Biol* **219**, 373–383.

Joly, W., Mugat, B. and Maschat, F. (2007). Engrailed controls the organization of the ventral nerve cord through frazzled regulation. *Dev Biol* **301**, 542–554.

Kumar, A., Fung, S., Lichtneckert, R., Reichert, H. and Hartenstein, V. (2009). Arborization pattern of Engrailed-positive neural lineages reveal neuromere boundaries in the Drosophila brain neuropil. *J. Comp. Neurol.* **517**, 87–104.

Liu, Z., Yang, X., Dong, Y. and Friedrich, M. (2006). Tracking down the "head blob": comparative analysis of wingless expression in the developing insect procephalon reveals progressive reduction of embryonic visual system patterning in higher insects. *Arthropod Struct Dev* **35**, 341–56.

Luan, Q., Chen, Q. and Friedrich, M. (2014). The Pax6 genes eyeless and twin of eyeless are required for global patterning of the ocular segment in the Tribolium embryo. *Developmental Biology* **394**, 367–381.

Martinez-Arias, A. and Lawrence, P. A. (1985). Parasegments and compartments in the Drosophila embryo. *Nature* **313**, 639–42.

Ntini, E. and Wimmer, E. A. (2011). Unique establishment of procephalic head segments is supported by the identification of cis-regulatory elements driving segment-specific segment polarity gene expression in Drosophila. *Dev Genes Evol* **221**, 1–16.

Oliver, G., Mailhos, A., Wehr, R., Copeland, N. G., Jenkins, N. A. and Gruss, P. (1995). Six3, a murine homologue of the sine oculis gene, demarcates the most anterior border of the developing neural plate and is expressed during eye development. *Development (Cambridge, England)* **121**, 4045–55.

Patel, N. H., Kornberg, T. B. and Goodman, C. S. (1989). Expression of engrailed during segmentation in grasshopper and crayfish. *Development* **107**, 201–12.

Posnien, N. and Bucher, G. (2010). Formation of the insect head involves lateral contribution of the intercalary segment, which depends on Tc-labial function. *Dev Biol* **338**, 107–16.

Posnien, N., Schinko, J. B., Kittelmann, S. and Bucher, G. (2010). Genetics, development and composition of the insect head – A beetle’s view. *Arthropod Structure & Development* **39**, 399–410.

Posnien, N., Koniszewski, N. and Bucher, G. (2011a). Insect Tc-six4 marks a unit with similarity to vertebrate placodes. *Dev Biol* **350**, 208–216.

Posnien, N., Koniszewski, N. D. B., Hein, H. J. and Bucher, G. (2011b). Candidate Gene Screen in the Red Flour Beetle Tribolium Reveals Six3 as Ancient Regulator of Anterior Median Head and Central Complex Development. *PLoS Genet* **7**, e1002418.

Posnien, N., Koniszewski, N. D. B., Hein, H. J. and Bucher, G. (2011c). Candidate Gene Screen in the Red Flour Beetle Tribolium Reveals Six3 as Ancient Regulator of Anterior Median Head and Central Complex Development. *PLoS Genetics* **7**, e1002416.

Puelles, L., Fernandez-Garre, P., Sanchez-Arrones, L., Garcia-Calero, E. and Rodriguez-Gallardo, L. (2005). Correlation of a chicken stage 4 neural plate fate map with early gene expression patterns. *Brain Res Brain Res Rev* **49**, 167–78.

Reversade, B., Kuroda, H., Lee, H., Mays, A. and De Robertis, E. M. (2005). Depletion of Bmp2, Bmp4, Bmp7 and Spemann organizer signals induces massive brain formation in Xenopus embryos. *Development* **132**, 3381–3392.

Schinko, J. B., Kreuzer, N., Offen, N., Posnien, N., Wimmer, E. A. and Bucher, G. (2008). Divergent functions of orthodenticle, empty spiracles and buttonhead in early head patterning of the beetle Tribolium castaneum (Coleoptera). *Dev Biol* **317**, 600–13.

Schlosser, G. (2014). Early embryonic specification of vertebrate cranial placodes. *Wiley Interdiscip Rev Dev Biol* **3**, 349–363.

Shimamura, K., Hartigan, D. J., Martinez, S., Puelles, L. and Rubenstein, J. L. (1995). Longitudinal organization of the anterior neural plate and neural tube. *Development (Cambridge, England)* **121**, 3923–33.

Siegler, M. V. and Jia, X. X. (1999). Engrailed negatively regulates the expression of cell adhesion molecules connectin and neuroglian in embryonic Drosophila nervous system. *Neuron* **22**, 265–276.

Siegler, M. V. and Pankhaniya, R. R. (1997). Engrailed protein is expressed in interneurons but not motor neurons of the dorsal unpaired median group in the adult grasshopper. *J Comp Neurol* **388**, 658–668.

Smith, F. W., Cumming, M. and Goldstein, B. (2018). Analyses of nervous system patterning genes in the tardigrade Hypsibius exemplaris illuminate the evolution of panarthropod brains. *Evodevo* **9**, 19.

Technau, G. M., Berger, C. and Urbach, R. (2006). Generation of cell diversity and segmental pattern in the embryonic central nervous system of Drosophila. *Dev Dyn* **235**, 861–9.

Ungerer, P. and Scholtz, G. (2008). Filling the gap between identified neuroblasts and neurons in crustaceans adds new support for Tetraconata. *Proc. Biol. Sci.* **275**, 369–376.

Urbach, R. (2007). A procephalic territory in Drosophila exhibiting similarities and dissimilarities compared to the vertebrate midbrain/hindbrain boundary region. *Neural Develop* **2**, 23.

Urbach, R. and Technau, G. M. (2003a). Segment polarity and DV patterning gene expression reveals segmental organization of the Drosophila brain. *Development* **130**, 3607–20.

Urbach, R. and Technau, G. M. (2003b). Molecular markers for identified neuroblasts in the developing brain of Drosophila. *Development* **130**, 3621–37.

van der Zee, M., Stockhammer, O., von Levetzow, C., Nunes da Fonseca, R. and Roth, S. (2006). Sog/Chordin is required for ventral-to-dorsal Dpp/BMP transport and head formation in a short germ insect. *Proc Natl Acad Sci U S A* **103**, 16307–12.

Wollesen, T., Rodríguez Monje, S. V., Todt, C., Degnan, B. M. and Wanninger, A. (2015). Ancestral role of Pax2/5/8 in molluscan brain and multimodal sensory system development. *BMC Evol Biol* **15**, 231.

Yang, X., Weber, M., Zarinkamar, N., Posnien, N., Friedrich, F., Wigand, B., Beutel, R., Damen, W. G., Bucher, G., Klingler, M., et al. (2009a). Probing the Drosophila retinal determination gene network in Tribolium (II): The Pax6 genes eyeless and twin of eyeless. *Dev Biol*.

Yang, X., Zarinkamar, N., Bao, R. and Friedrich, M. (2009b). Probing the Drosophila retinal determination gene network in Tribolium (I): The early retinal genes dachshund, eyes absent and sine oculis. *Dev Biol* **333**, 202–14.
